# Responses of wheat kernel weight to diverse allelic combinations under projected climate change conditions

**DOI:** 10.3389/fpls.2023.1138966

**Published:** 2023-03-14

**Authors:** Keyi Wang, Liping Shi, Bangyou Zheng, Yong He

**Affiliations:** ^1^ Institute of Environment and Sustainable Development in Agriculture, Chinese Academy of Agricultural Sciences, Beijing, China; ^2^ Commonwealth Scientific and Industrial Research Organisation (CSIRO) Agriculture and Food, Queensland Biosciences Precinct, St. Lucia, QLD, Australia

**Keywords:** allelic combination, climate change, thousand-kernel weight, winter wheat (*Triticum aestivum L*.), APSIM-Wheat model

## Abstract

**Introduction:**

In wheat, kernel weight (KW) is a key determinant of grain yield (GY). However, it is often overlooked when improving wheat productivity under climate warming. Moreover, little is known about the complex effects of genetic and climatic factors on KW. Here, we explored the responses of wheat KW to diverse allelic combinations under projected climate warming conditions.

**Methods:**

To focus on KW, we selected a subset of 81 out of 209 wheat varieties with similar GY, biomass, and kernel number (KN) and focused on their thousand-kernel weight (TKW). We genotyped them at eight kompetitive allele-specific polymerase chain reaction markers closely associated with TKW. Subsequently, we calibrated and evaluated the process-based model known as Agricultural Production Systems Simulator (APSIM-Wheat) based on a unique dataset including phenotyping, genotyping, climate, soil physicochemistry, and on-farm management information. We then used the calibrated APSIM-Wheat model to estimate TKW under eight allelic combinations (81 wheat varieties), seven sowing dates, and the shared socioeconomic pathways (SSPs) designated SSP2-4.5 and SSP5-8.5, driven by climate projections from five General Circulation Models (GCMs) BCC-CSM2-MR, CanESM5, EC-Earth3-Veg, MIROC-ES2L, and UKESM1-0-LL.

**Results:**

The APSIM-Wheat model reliably simulated wheat TKW with a root mean square error (RMSE) of < 3.076 g TK^-1^ and R^2^ of > 0.575 (*P* < 0.001). The analysis of variance based on the simulation output showed that allelic combination, climate scenario, and sowing date extremely significantly affected TKW (*P* < 0.001). The impact of the interaction allelic combination × climate scenario on TKW was also significant (*P* < 0.05). Meanwhile, the variety parameters and their relative importance in the APSIM-Wheat model accorded with the expression of the allelic combinations. Under the projected climate scenarios, the favorable allelic combinations (TaCKX-D1b + Hap-7A-1 + Hap-T + Hap-6A-G + Hap-6B-1 + H1g + A1b for SSP2-4.5 and SSP5-8.5) mitigated the negative effects of climate change on TKW.

**Discussion:**

The present study demonstrated that optimizing favorable allelic combinations can help achieve high wheat TKW. The findings of this study clarify the responses of wheat KW to diverse allelic combinations under projected climate change conditions. Additionally, the present study provides theoretical and practical reference for marker-assisted selection of high TKW in wheat breeding.

## 1 Introduction

Wheat is one of the most important staple crops and constitutes ~21% of the global food crop production (FAOSTAT, 2021). By 2050, the global demand for wheat is expected to increase at an annu al rate of 1.7%; however, the wheat grain yield (GY) is expected to increase by only 1.1% per year at that time (Ray et al., 2013). The thousand-kernel weight (TKW) is a key determinant of winter wheat GY (Li et al., 2022). Kernel number per spike and spike number per unit area (ha) (SN) are becoming increasingly stable as wheat genomics rapidly develops (Fischer, 2008). Previous studies have shown that increasing TKW effectively increases GY (Qin et al., 2015; Duan et al., 2020).

Grain formation comprises photosynthate production, transport, accumulation, and solidification (Serrago et al., 2013). Kernel weight (KW) depends mainly on grain size (volume) and degree of filling (plumpness). Larger grains and faster, earlier, and longer grain filling are associated with higher KW (Xie et al., 2015). The grain filling rate divides KW formation into gradual, rapid, and slow increase periods (Wang and Shangguan, 2015). The gradual increase period is the “reservoir establishment” stage. Temperature, photosynthetic nutrition, and phytohormone levels cooperatively induce endosperm cells to form the grain through division and growth. The number of endosperm cells determines the grain size (Schnyder and Baum, 1992). Grain filling slowly accelerates and grain dry matter accumulation accounts for 13–20% of the total mature KW during the gradual increase period. The rapid increase period is the “reservoir formation” stage. At this stage, the assimilates required for grain filling originate mainly from the photosynthetic products formed after flowering and the mobilization of soluble reserves from the nutritional organs before and after flowering. Genotype, climatic conditions, and nutrient supply levels influence this process (Ehdaie et al., 2008). During the rapid increase period, the rate of grain filling increases considerably and grain dry matter accumulation is highest and accounts for 52–57% of the mature KW. The slow increase period is the “reserve solidification” stage. Senescent plant organs supply assimilates as their structural macromolecules degrade and are transported to the developing grains at the late plant growth stage (Distelfeld et al., 2014). During the slow increase period, the grain filling rate gradually decreases, grain filling eventually stops, the grain gradually matures, and dry matter accumulation accounts for 12–20% of the mature KW.

Wheat KW is influenced by both genetic and environmental factors (Brinton and Uauy, 2019). Kernel weight is a quantitative trait regulated by several different genes with a stable phenotype and medium to high heritability (0.6–0.8); however, it is highly susceptible to environmental factors (Miao et al., 2022). Research has revealed that breeding for superior allele aggregation, which in turn regulates and modifies gene expression as well as biochemical and metabolic pathways, is an important way to increase KW. Qin et al. (2015) conducted a linear regression analysis of >1,850 Chinese wheat varieties produced since the 1920s, which revealed that the average TKW increased from 30.16 g TK^−1^ in the 1920s to 38.43 g TK^−1^ in the 2010s (Qin et al., 2015). Furthermore, the use of molecular marker technology greatly enhanced the improvement of thousand grain weight traits. Wheat *TaSus1-7A* and *TaSus2-7B* encode sucrose synthases and are positively correlated with dry matter accumulation during wheat development (Hou et al., 2014). Auxin regulates cell elongation, expansion, and division during grain filling and affects grain size and weight. *TaGW2-6A* negatively regulates wheat KW by inhibiting auxin biosynthesis (Geng et al., 2017; Li et al., 2017). Environmental factors, particularly temperature, also play vital roles in KW. Temperature substantially altered KW by affecting wheat phenological development, grain filling rate, and duration (Li et al., 2022; Li et al., 2022).Elevated temperatures accelerate the growth and development of wheat, shorten the wheat nutritional growth stage, and reduce dry matter accumulation in the nutritional organs before flowering (Juknys et al., 2017). Grain filling has a heat duration of approximately 700 growing degree-days (GDD). Hence, grain filling is shortened if the ambient temperature is particularly high during this period (Zhao et al., 2007). At temperatures between 20°C and 30°C, the grain filling rate slightly increases with temperature but does not compensate for the reduction in grain filling duration. At temperatures > 30°C, the grain filling rate slows down in part because of thermal damage to the plant organs and accelerated plant senescence (Wardlaw and Moncur, 1995; Lobell et al., 2012).

A suitable sowing date is an important management strategy for the modulation of winter wheat KW. Tillering development, environmental conditions, and the timing and duration of the nutritional and reproductive stages of wheat growth and development may vary with sowing date. Thus, adjustments to the sowing date can alter photosynthesis and nutrient transport during wheat growth and development and, therefore, KW accumulation (Silva et al., 2014; Wahid et al., 2017; Liu et al., 2021). An appropriate sowing date promotes tillering development, increases light interception, photosynthesis, and dry matter accumulation, and improves transport efficiency (Dueri et al., 2022). However, sowing that is too early or too late weakens tillering, lowers the grain filling rate and duration, and decreases KW (Shukla et al., 2022).

Previous research focused mainly on the impact of climate change on wheat GY but did not explore its influences on KW. The latter is a key determinant of GY potential and future climate adaptability (Tian et al., 2014; Tack et al., 2015; Zhao et al., 2016; Shew et al., 2020). Process-based crop models are often used to study the effects of genetics, field management, and their interactions on wheat growth and development as well as the impact of climate warming on wheat productivity. Briak and Kebede (2021) used their experimental data to calibrate and evaluate the Agricultural Production Systems Simulator (APSIM-Wheat) model and demonstrated its accuracy. Their simulation experiments showed that optimal sowing dates for suitable wheat varieties improve crop adaptation to climate warming. Here, we implemented the APSIM-Wheat model to elucidate the mechanisms by which KW responds to genotype and sowing date and improve its KW under climate warming. Out of a pool of 209 wheat varieties, we selected 81 with similar GY, biomass, and kernel number (KN). We used these KW, GY, and KN values to calibrate and evaluate the APSIM-Wheat model and then applied the evaluated model to simulate KW under eight allelic combinations (81 wheat varieties), seven sowing dates, and two shared socioeconomic pathways (SSPs). The objectives were to mitigate the negative impact of future climate warming on KW and achieve high KW. The results of the simulation enabled the identification of adaptive sowing dates and favorable allelic combinations for KW improvement. The findings of this study help clarify the physiological and ecological mechanisms by which KW responds to genotype and sowing date under climate warming and provide theoretical and practical references for high KW development in winter wheat.

## 2 Materials and methods

### 2.1 Field conditions

Field experiments were conducted in 2018–2019 and in 2019–2020 at the Xinxiang Comprehensive Experimental Station of the Chinese Academy of Agricultural Sciences, Henan Province, China (35°18′N, 113°51′E, 78 m a.s.l.). The region has a warm, temperate, semi-humid climate. The brown soil of the region had pH and bulk density of 7.11 and 1.38 g cm^-3^, respectively. The water, organic carbon, and total nitrogen levels were 36.1%, 1.7 g kg^-1^, and 1.11 g kg^-1^, respectively. In 2018–2019 and 2019–2020, the accumulated temperatures were 2559.3 °C and 2437.1 °C and the precipitation levels were 105.5 mm and 110.6 mm, respectively (Ma et al., 2021).

### 2.2 Experimental design

Two hundred and nine varieties were selected among natural wheat populations and Huaimai 40 (stable) and Zhengmai 7698 (sensitive) served as the control varieties. They were sown on October 8, 2018 and October 23, 2019, respectively, and harvested on June 6, 2019 and June 2, 2020, respectively. The experiment was repeated twice using a randomized complete block design. The sowing density, row spacing, sowing depth, and plot size were 270 plants m^-2^, 10 cm, 5 cm, and 4.2 m^2^ (3 m × 1.4 m), respectively. Details of water and fertilizer management during the two growing seasons are listed in **Table 1**. The SN, KN, GY, and TKW were measured during two growing seasons. Two random samples were taken, after the grain filling stage, from each sample frame (0.5 × 0.5 m) within the test plots (excluding edge areas). The SN in the sample frames was recorded, and the average value was obtained. Before the wheat maturity, 20 representative ears of wheat were randomly selected from each plot and threshed to obtain the average KN. After the wheat matured, it was threshed, dried, and weighed to obtain the GY per unit area (ha). Representative kernels (1000) were then selected and weighed to obtain the TKW. Pest and weed control were implemented in accordance with local productive field standards.

**Table 1:** On-farm management of 209 wheat varieties during the 2018-2019 and 2019-2020 growing seasons.

Growing seasons	Date	Irrigation (mm)		Fertilization (kg/ha)	
2018-2019	10/4 (Before sowing)			Humic acid urea 12.5 + Di-ammonium phosphate 30 + KCl 7.5	
	12/15 (Overwintering)	180			
	2/27 (Elongation)			N26-P0-K4 15	
	3/7 (Elongation)	120			
	5/17 (Grouting)	120			
2019-2020	10/22 (Before sowing)			Humic acid urea 11 + Controlled-release urea 4 + Mono-ammonium phosphate 30 +Potassium 5	
	12/24 (Overwintering)	150			
	12/28 (Overwintering)			N26-P0-K4 10	
	3/1 (Elongation)			N26-P0-K4 10	
	3/12 (Elongation)	120			
	4/27 (Grouting)	120			

### 2.3 Materials

**Figure 1:**
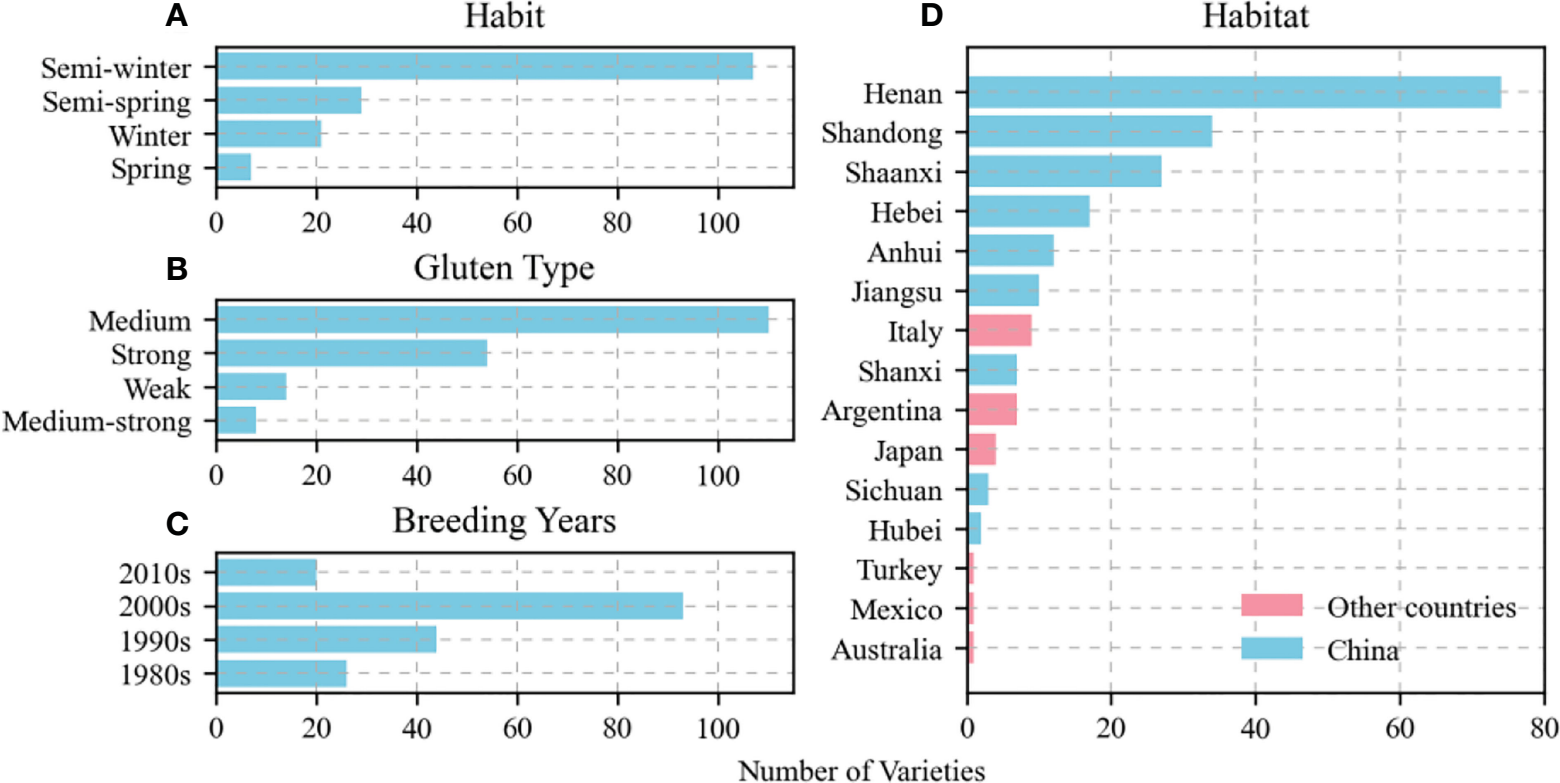
Distribution of tested varieties in terms of habit **(A)**, gluten type **(B)**, breeding year **(C)**, and habitat particularly among nine provinces in China **(D)**.

The present study included 209 wheat varieties sampled from different habits (winter, semi-winter, weak-spring, or spring), gluten content types (strong, medium-strong, medium, or weak), breeding years (1980s–2010s), and habitats (China or other countries). The materials were abundant and widely distributed. The medium gluten type had the highest proportion (59.14%) followed by the strong gluten (29.03%) and the weak and medium-strong gluten types (7.53% and 4.30%, respectively). Additionally, the proportion of the semi-winter type was the highest (65.24%) followed by the weak-spring type (17.68%) and the winter and spring types (12.80% and 4.27%, respectively). The crops cultivated in the 2000s had the highest proportion (50.82%) followed by those grown in the 1990s (24.04%) and those raised in the 1980s and the 2010s (14.21% and 10.93%, respectively). The Chinese varieties accounted for the highest proportion (89.00%) and were distributed across nine provinces. The varieties were distributed across six countries and accounted for only 11.00% of the total (**Figure 1**).

### 2.4 Genotyping

DNA was extracted from the leaves of the wheat seedlings using the high-salt/low-pH method (Guillemaut and Maréchal-Drouard, 1992) and amplified by PCR. A kompetitive allele-specific polymerase chain reaction (KASP) fluorescence detector was used to determine the genotypes for seven functional genes that regulate KW (*TaCKX-D1, TaGASR7-A1, TaSus1-7A, TaSus1-7B, TaGS5-A1, TaGW2-6A*, and *TaKG2-6B*), considerably affect its characteristics, and are associated with KASP markers (Rasheed et al., 2019). The primer sequences and amplification conditions for each gene are described in **Supplementary Table 1**. KASP detection revealed 209 varieties with 41 KW allelic combinations (**Supplementary Table 2**). Their wide variety ensured genotypic diversity in this study.

### 2.5 Data collection

Meteorological, soil, field management, and observation data were collected to calibrate and evaluate the parameters of the APSIM-Wheat model. The meteorological data included daily maximum and minimum temperatures, daily sunshine hours, and daily precipitation at the Xinxiang Agrometeorological Station between 1961 and 2020. The phenological stage dataset included emergence, three-leaf stage, tillering, elongation, booting, heading, anthesis, medium milk, and maturity at the Xinxiang station between 2001 and 2013. The soil data used to run the model and calibrate the soil parameters were obtained from Zain et al. (2021) and included soil water and nitrogen distribution during the winter wheat growth periods between 2017 and 2019. The field observation data used to calibrate and evaluate TKW, KN, and GY were derived from the phenotypic observation data (Normalized Difference Vegetation Index (NDVI), TKW, GY, KN, and SN) of the 209 varieties under different managements at the Xinxiang station between 2018 and 2020. They also included data for the shared socioeconomic pathways SSP2-4.5 and SSP5-8.5 of the five global climate models in the Coupled Model Intercomparison Project Phase 6, namely, BCC-CSM2-MR (China), CanESM5 (Canada), EC-Earth3-Veg (Europe), MIROC-ES2L (Japan), and UKESM1-0-LL (UK). Specific on-site farm managements are listed in **Supplementary Table 3**. The physicochemical properties of the various soil layers are listed in **Supplementary Table 4**.

### 2.6 Variety screening and statistical analysis

**Figure 2:**
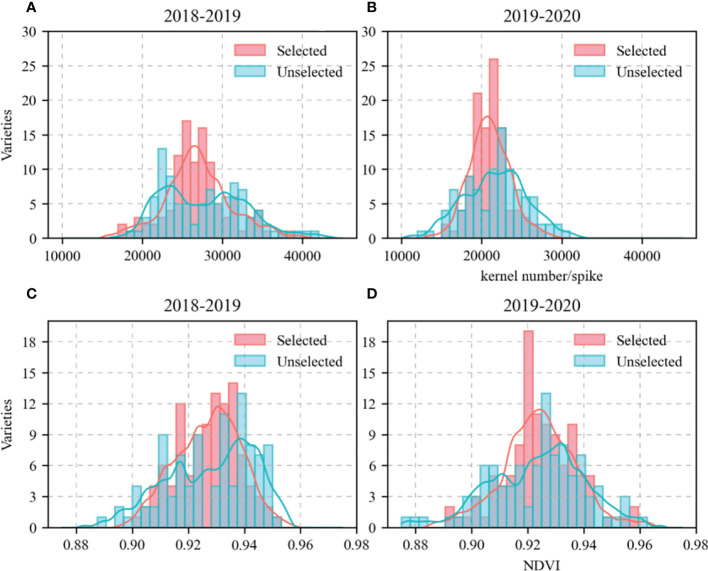
kernel number (KN) and Normalized Difference Vegetation Index (NDVI) of 209 wheat varieties in growing season. **(A, B)**, KN; **(C, D)**, NDVI. Red columns represent varieties with similar KN and NDVI. Blue columns represent varieties with larger differences in KN and NDVI.

**Figure 3:**
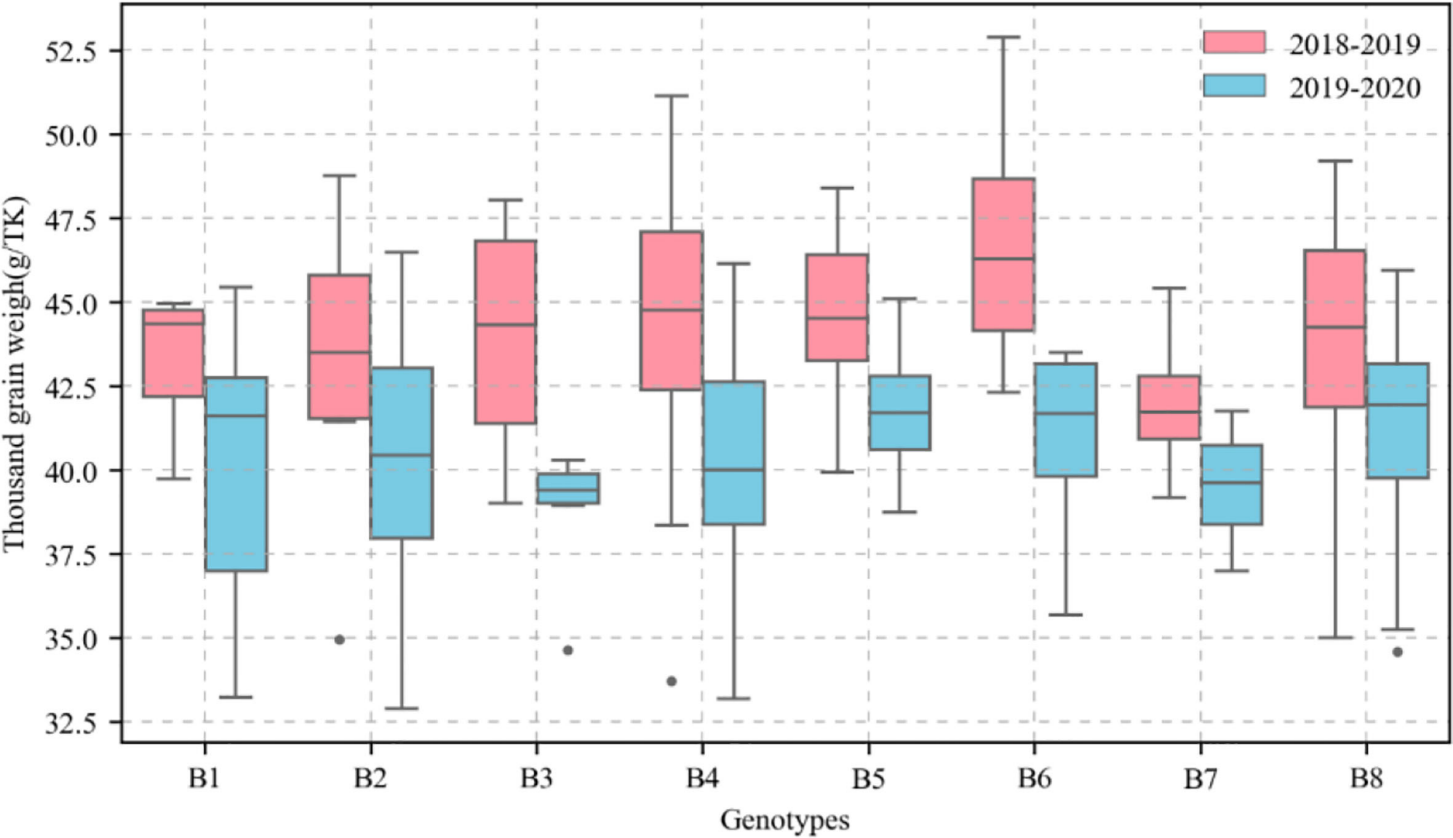
Distribution of thousand-kernel weight (TKW) of different genotypes of wheat in different growing seasons. Red box represents TKW in 2018-2019 growing season. Blue box represents TKW in 2019-2020 growing season.

As the study focused on the mechanisms by which KW responds to sowing date and genotype, 209 wheat varieties were screened here. First, it was assumed that varieties with KN and flowering period NDVI in close proximity had other genetic factors near the KW-regulating gene. Hence, varieties with similar KN (range < 5,000) and flowering period NDVI (range < 0.025) were selected. The data analysis was verified using the Kruskal–Wallis test. Eighty-one varieties met the foregoing criteria and did not significantly differ in terms of KN or NDVI. The Kruskal–Wallis test was run using the “npmc” package in R v. 3.6.2 (http://www.R-project.org/). The KN and NDVI for the 81 selected and 128 unselected varieties during their growth seasons are shown in **Figure 2**. The allelic combinations and percentages are listed in **Table 2**. The KW distributions for the selected 81 varieties in eight allelic combinations are shown in **Figure 3**.

**Table 2:** Allelic combinations for kernel weight (B, eight combinations).

Haplotype	TaCKX-D1	TaSus1-7A	TaSus1-7B	TaGW2-6A	TaGW2-6B	TaGASR7-A1	TaGS5-A1	Percent/%
B1	TaCKX-D1b	Hap-7A-1	Hap-T	Hap-6A-A	Hap-6B-1	Hlc	Alb	11.10
B2	TaCKX-D1b	Hap-7A-1	Hap-T	Hap-6A-A	Hap-6B-1	Hlg	Ala	13.60
B3	TaCKX-D1b	Hap-7A-1	Hap-T	Hap-6A-A	Hap-6B-2	Hlg	Ala	7.40
B4	TaCKX-D1b	Hap-7A-1	Hap-T	Hap-6A-A	Hap-6B-1	Hlg	Alb	33.30
B5	TaCKX-D1b	Hap-7A-1	Hap-T	Hap-6A-A	Hap-6B-2	Hlg	Alb	8.60
B6	TaCKX-D1b	Hap-7A-1	Hap-T	Hap-6A-G	Hap-6B-1	Hlg	Alb	7.40
B7	TaCKX-D1b	Hap-7A-2	Hap-T	Hap-6A-A	Hap-6B-2	Hlg	Ala	4.90
B8	TaCKX-D1b	Hap-7A-2	Hap-T	Hap-6A-A	Hap-6B-1	Hlg	Alb	13.60

### 2.7 Sensitivity analyses of KW, KN, and GY parameters in the APSIM-Wheat model

The eFAST method was used in this study to elucidate the relationships among the variety and ecotype parameters and accurately simulate KW, KN, and GY. The eFAST method has been used to assess the sensitivity of various models (Campolongo et al., 2000) and was applied in four steps.

(1) The parameters to be analyzed in the APSIM-Wheat model were selected and their range of values was determined. In the present study, eight variety and 33 ecotype parameters were selected (**Supplementary Table 5**) and their upper and lower limits were set.
(2) Monte Carlo sampling was used to generate a random parameter sample set. Here, all parameters were sampled with a uniform distribution implemented in Simlab software. In eFAST, the number of parameters sampled was > 65-fold greater that the number of parameters to be validated, that is, sample number ≥ parameters number × 65. Thus, 128 samples × 41 (model parameters) × two years × two treatments = 20,992 model parameters.
(3) The random parameter samples were used as the input for the APSIM-Wheat model and the simulation results were generated. Python v. 3.9.15 (https://www.python.org/) was used to write a program that sequentially replaces the 41 parameters in the APSIM Wheat.xml file and calls the executable file (APSIM.exe) of the APSIM-Wheat model.
(4) Simlab software was then used to perform parametric first-order and global sensitivity analyses of wheat KW, KN, and GY (Giglioli and Saltelli, 2000). The model simulation results were organized into a standard file input format recognizable by Simlab. The latter automatically calculated the first-order and global sensitivity indices of the input parameters.

Both the first-order sensitivity and the global sensitivity indices are used to assess the sensitivity of the system to the input parameters. However, the former only considers the effect of a single input parameter on the output parameter, while the latter considers the interaction among multiple input parameters. The closer the value of the sensitivity index is to 1, the greater will be the influence of this input parameter on the output parameter; the closer the value is to 0, the smaller will be the influence on the output parameter.

### 2.8 Model calibration and evaluation

The phenological, soil, variety, and ecotype model parameters directly and indirectly affecting KW formation were calibrated and evaluated. The wheat phenological parameters were calibrated and evaluated *via* batch parameter adjustment (**Supplementary Table 6**). The simulation was conducted under water- and nitrogen stress-free conditions. The physical properties were then calibrated and evaluated (**Supplementary Table7**). Based on the results of the parameter sensitivity analysis, the variety and ecotype parameters related to KN were then calibrated and evaluated. The range of parameter values was obtained for the optimally simulated KN results for the 209 varieties. The variety and ecotype parameters related to KW and GY were then calibrated and evaluated. The range of parameter values was obtained for the optimally simulated GY and KW results for the 209 varieties (**Supplementary Table 8**). Root mean square error (RMSE) was used to determine the total difference between the observed and simulated values. Linear regression (R^2^) between the measured and simulated values was used to evaluate model performance. Consistency (D) between the measured and simulated values was also determined.

### 2.9 Simulation scheme and statistical analysis

Simulations were run to (1) explore the responses of KW to sowing date and genotype under the background of climate warming, and (2) screen adaptive sowing dates and favorable allelic combinations that enhance KW. The APSIM-Wheat model was used to simulate KW under various sowing dates, allelic combinations, and climate scenarios as follows:

(1) Historical sowing date data from the Xinxiang station between 2001 and 2012 were used to calculate the average value and the latter was then set as the current sowing date (October 11; day of year (DOY) 284). The sowing date period was from October 5 to October 23 and the specific sowing dates were October 5 (DOY 278), October 8 (DOY 281), October 11 (DOY 284), October 14 (DOY 287), October 17 (DOY 290), October 20 (DOY 293), and October 23 (DOY 296).
(2) Eighty-one varieties were selected and divided into eight allelic combinations based on seven KW functional genes.
(3) The SSP2 and SSP5 scenarios were selected and the BCC-CSM2-MR, CanESM5, EC-Earth3-Veg, MIROC-ES2L, and UKESM1-0-LL global climate models were used in the baseline (1991–2020) and future (2031–2060) periods.

R v. 3.6.2 (http://www.R-project.org/) was used to analyze and test the significance of the differences in KW under various sowing dates, allelic combinations, and climate scenarios. The AOV function in the “stats” package of R was used to perform the analysis of variance (ANOVA) and the lsd.test function in the “multcomp” package of R was used to perform the analysis of multiple comparison.

## 3 Results

### 3.1 Sensitivity analysis of the model parameters affecting KW, GY, and KN

The first-order and global sensitivity indices of the factors affecting wheat KW, GY, and KN were consistent. Of the 41 parameters affecting KW, the variety parameters “max_grain_size” and “grains_per_gram_stem” had first-order sensitivity indices > 0.2 and global sensitivity indices > 0.4. The ecotype parameters “y_swdef_leaf” and “fr_lf_sen_rate” had first-order sensitivity indices > 0.05 and global sensitivity indices > 0.1. The ecological parameter “node_sen_rate” had a first-order sensitivity index > 0.025 and a global sensitivity index > 0.1. The ecotype parameters “y_rue” and “eo_crop_factor_default” had global sensitivity indices > 0.05. Of the 41 parameters affecting GY, the variety parameters “max_grain_size”, and “grains_per_gram_stem” and the ecotype parameters “y_swdef_leaf”, “oxdef_photo”, “node _sen_rate”, and “y_swdef_pheno_flowering” had first-order sensitivity indices > 0.05 and global sensitivity indices > 0.1. Of the 41 parameters affecting KN, the species parameter “grains_per_ gram_stem” and the ecotype parameters “y_swdef_leaf”, “oxdef_photo”, and “y_swdef_ pheno_flowering” had first-order sensitivity indices > 0.05. The species parameter “grains_per_gram_stem” and the ecotype parameters “y_swdef_ leaf”, “initial_root_depth”, “oxdef_photo”, and “y_rue” had global sensitivity indices > 0.1.

### 3.2 Phenological model parameter calibration and evaluation

**Figure 4:**
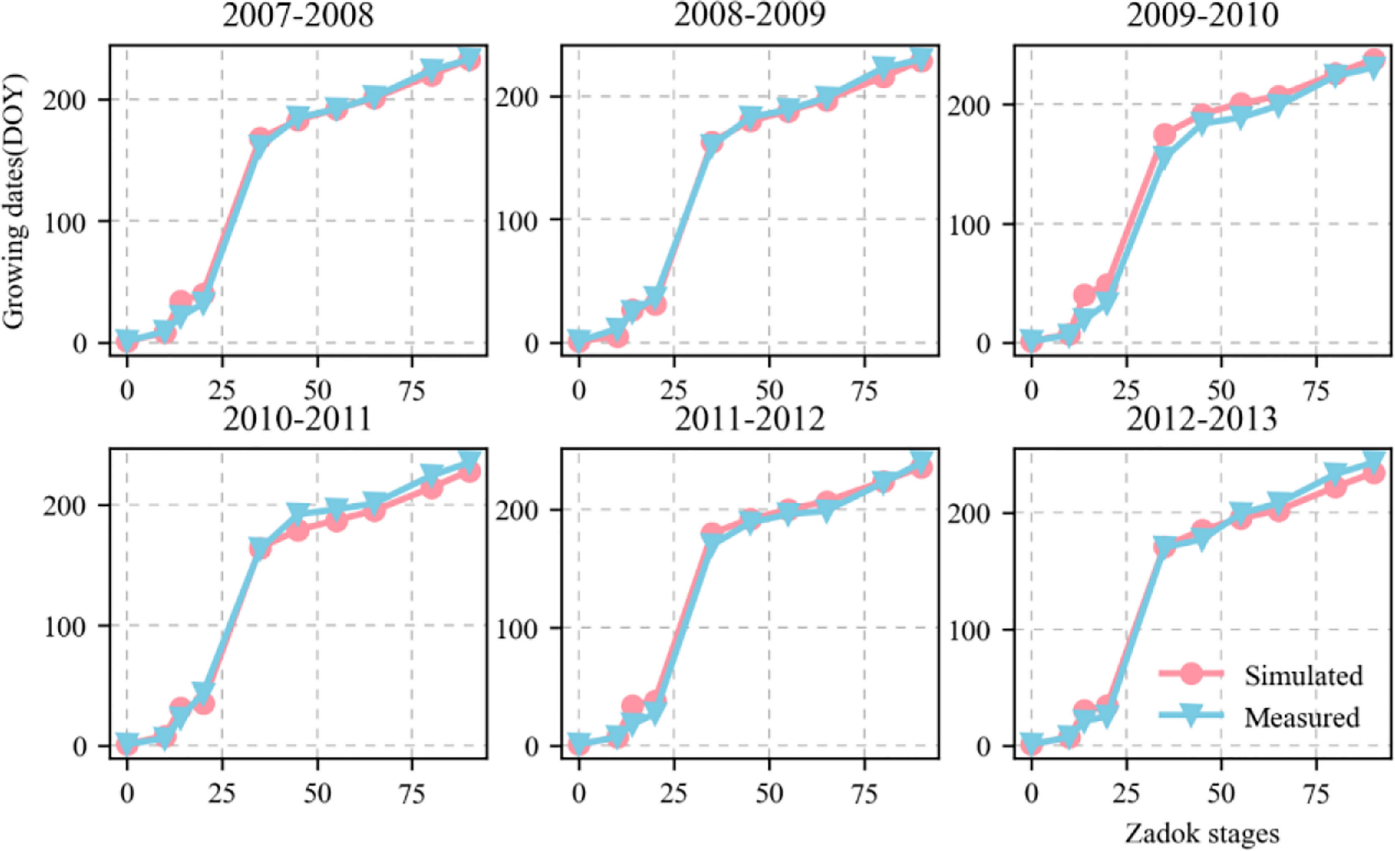
Comparison between measured and simulated values of wheat growth stages (Zadoks growth stage) including emergence (Z10), three leaves (Z14), tillering (Z20), elongation (Z35), booting (Z45), heading (Z55), anthesis (Z65), medium milk (Z80), and maturity (Z100). Red circles and blue triangles represent simulated and measured values, respectively. Zadoks growth stage is sourced from Zadoks et al. (1974). .

**Figure 5:**
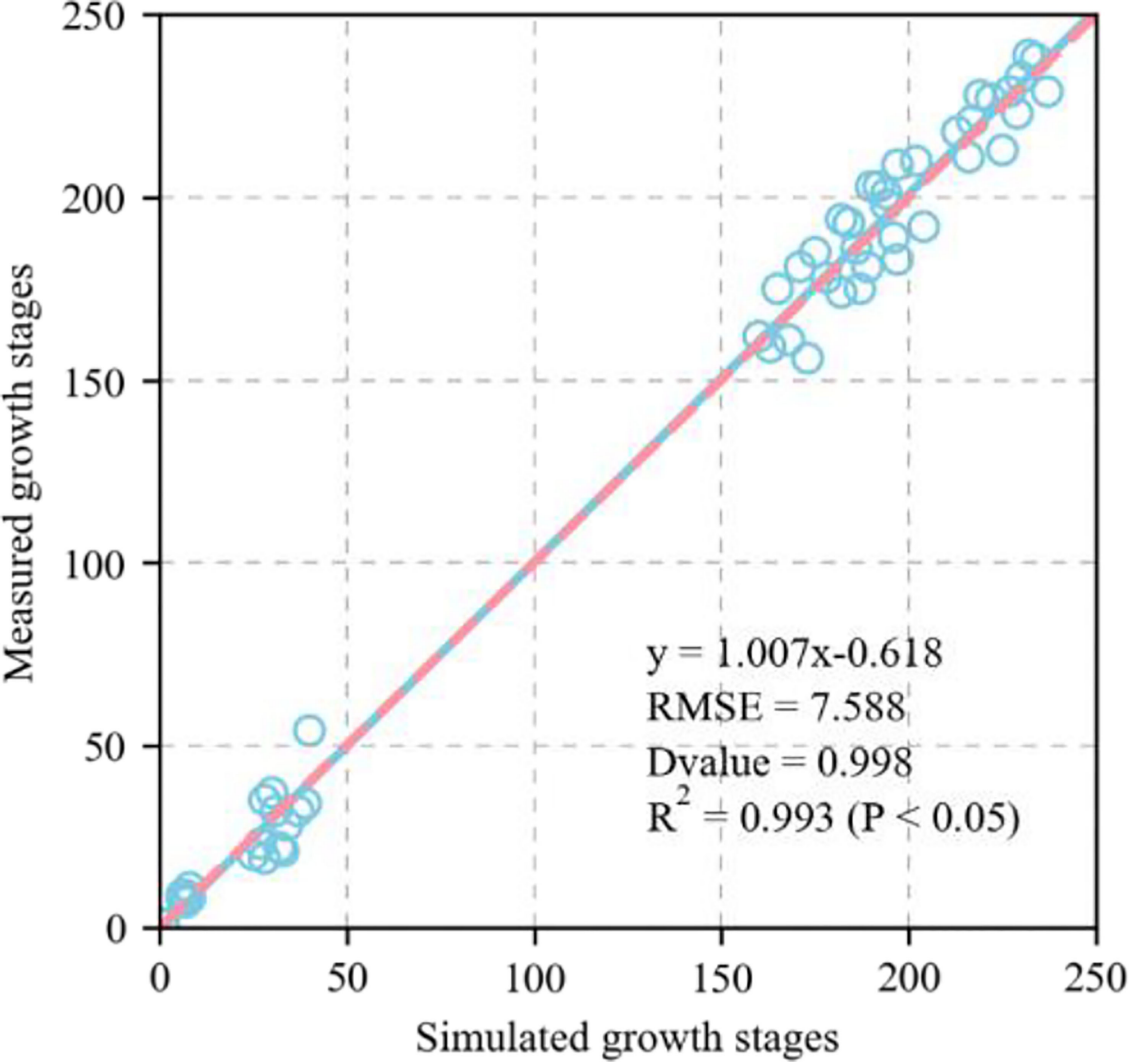
Evaluation of APSIM-Wheat model for modelling wheat growth stages (Zadoks growth stage) (1:1, dashed line), including emergence (Z10), three leaves (Z14), tillering (Z20), elongation (Z35), booting (Z45), heading (Z55), anthesis (Z65), medium milk (Z80), and maturity (Z100), at Xinxing station for evaluation dataset (2001–2007) (1:1, dashed line). Blue circle represents simulated and measured values. Straight line represents regression line of fitting equation between measured and simulated value. RMSE is root mean square error which is used to measure deviation between observed and measured value. D-value is used to assess consistency between observed and measured value. R^2^ is used to describe degree of fitting of regression line to observed value. The closer R^2^ is to 1, the better the fitting. P < 0.05 means difference was highly significant. Zadoks growth stage is sourced from Zadoks et al. (1974).

**Figure 4** shows the validation of the simulated phenological growth stages of wheat including emergence, three leaves, tillering, elongation, booting, heading, anthesis, medium milk, and maturity, at the experimental base in Xinxiang, Henan Province, China between 2007 and 2013. The simulated and measured phenological stages were consistent. We compared the simulated and measured values for the growth stages between 2001 and 2007 to evaluate the parameters independently. The fitted linear equation was y = 1.007x - 0.618 and its slope was ~1. As RMSE = 7.5 d, the deviation between the measured and simulated values for the phenological stage was 8 d. The D values calculated from the measured and simulated values was 0.998. The R^2^ was 0.993 and P < 0.05 (**Figure 5**).

### 3.3 Soil model parameter calibration and evaluation

**Figure 6:**
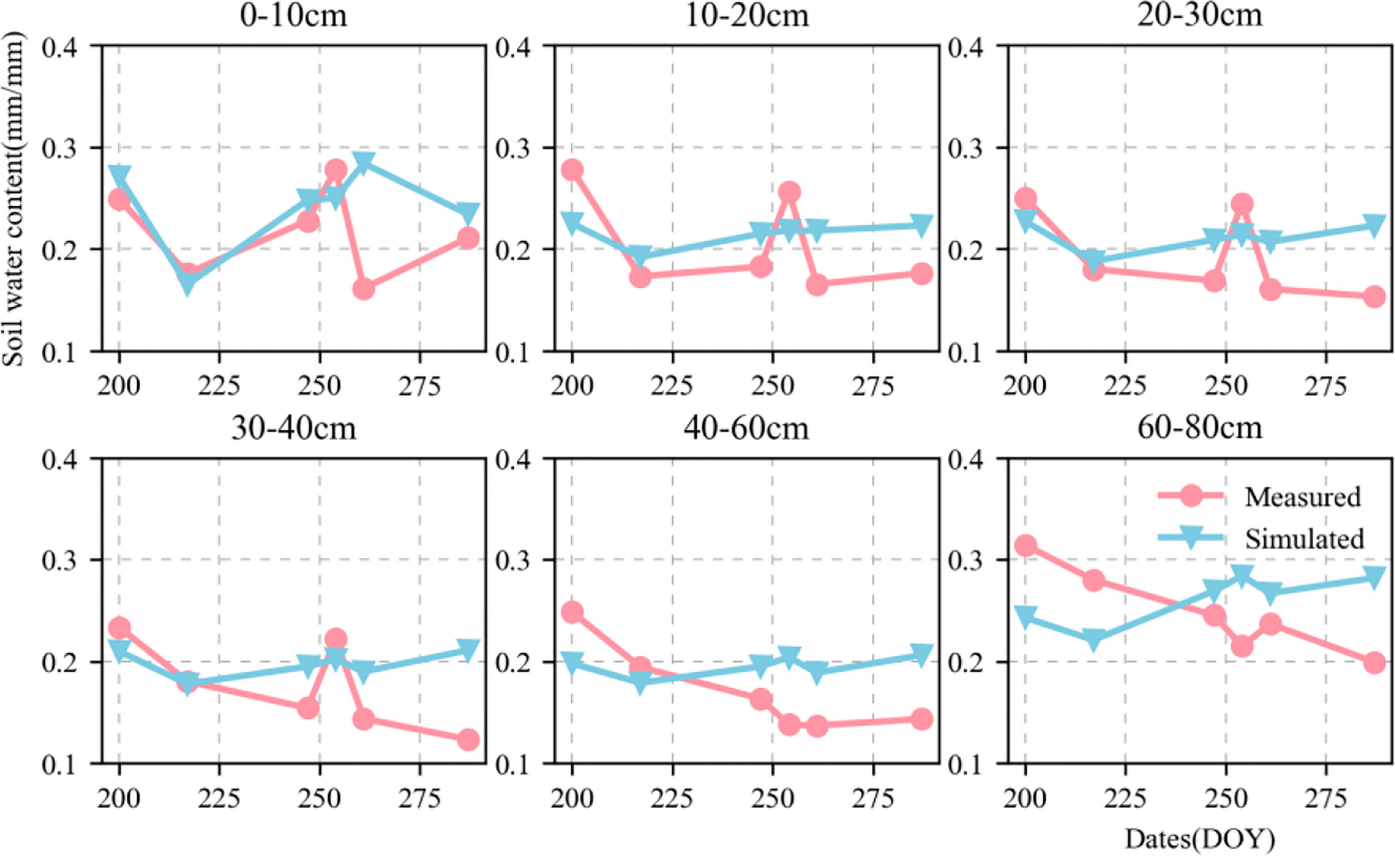
Dynamics of simulated (blue triangle) and measured (red circle) soil moisture content value in 0–80-cm soil profile at Xinxiang site from 2018 to 2019.

**Figure 7:**
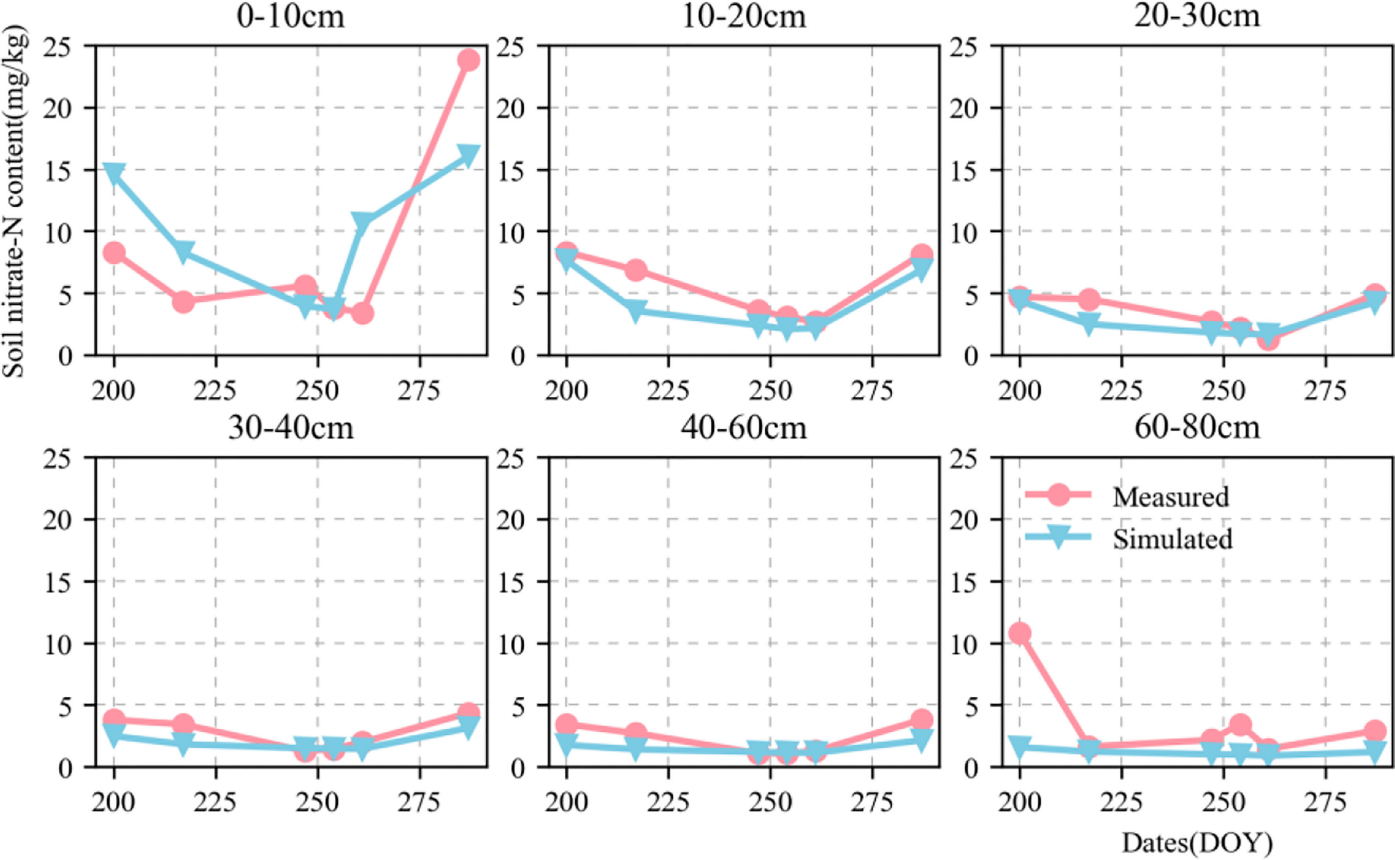
Dynamics of simulated (blue triangle) and measured (red circle) soil nitrate content value in 0–80-cm soil profile at Xinxiang site from 2018 to 2019.

**Figure 8:**
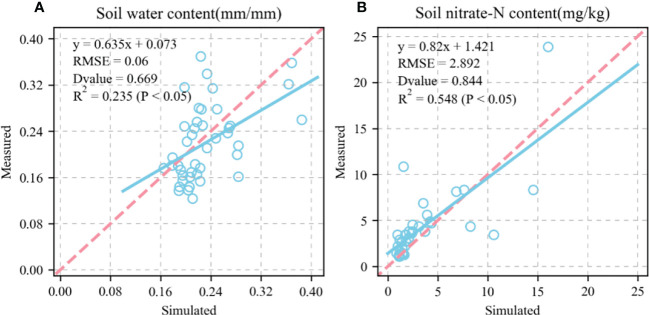
Comparison between simulated and measured soil water content and soil nitrate content (1:1, dashed line). **(A)**, soil water content; **(B)**, soil nitrate content. Blue circle represents simulated and measured values. Straight line represents regression line of fitting equation between measured and simulated value. RMSE is root mean square error which is used to measure deviation between observed and measured value. D-value is used to assess consistency between observed and measured value. R^2^ is used to describe degree of fitting of regression line to observed value. The closer is to 1, the better the fitting. P < 0.05 means difference was highly significant.

**Figures 6**, **7** show the validation of simulated water and nitrate nitrogen content in the 0–80-cm soil layer at the experimental base in Xinxiang, Henan Province, China between 2017 and 2018. The simulated results for the water and nitrate nitrogen content were good for the surface soil layer but poor for the deep soil layer. This could be owing to a lack of accurately measured soil data. Using data obtained from the literature may not fully reflect the actual local conditions, resulting in inaccurate simulation results for the deep soil layer. We compared the simulated and measured values for the soil water and nitrate nitrogen content in 2018–2019 to evaluate the parameters independently. The fitted linear equations were y = 0.635x + 0.073 and y = 0.82x + 1.421 (**Figure 8**). The RMSE were 0.06 mm mm^-1^ and 2.892 mg kg^-1^, respectively, and the D values were 0.669 and 0.844, respectively. The R^2^ were 0.235, 0.548 (P < 0.05), respectively.

### 3.4 Model parameter calibration and evaluation for KW, GY, and KN determinations

**Figure 9:**
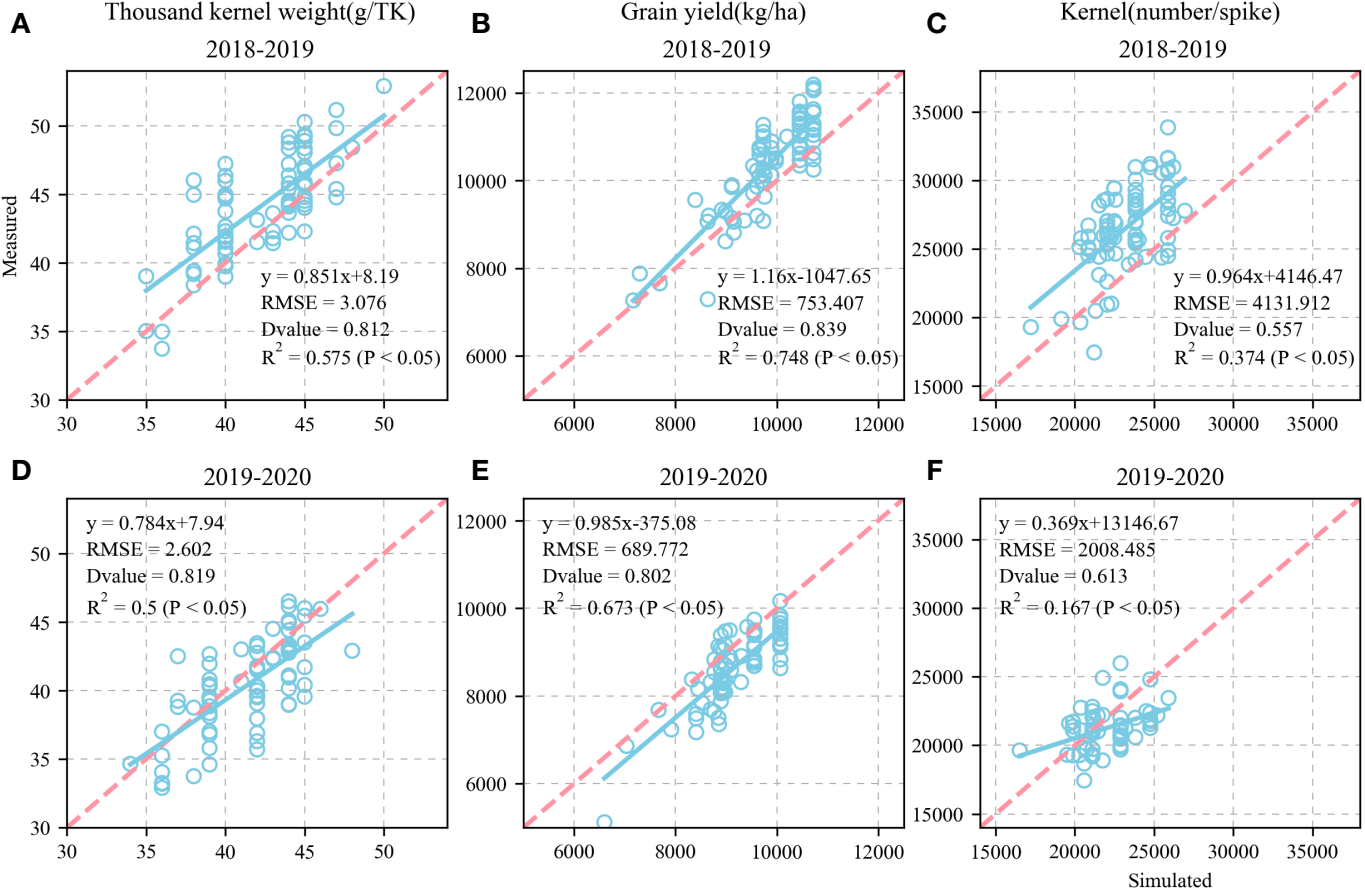
Calibration and evaluation of APSIM-Wheat model for modelling thousand-kernel weight (TKW), grain yield (GY) and kernel number (KN) and (1:1,dashed line) of 81 wheat varieties at Xinxing station for calibration dataset (2018-2019) and evaluation dataset (2019-2020) (1:1, dashed line). Comparison of observed and simulated thousand-kernel weight (TKW) **(A)**, grain yield (GY) **(B)**, and kernel number (KN) **(C)** for calibration datasets. Comparison of observed and simulated thousand-kernel weight (TKW) **(D)**, grain yield (GY) **(E)**, and kernel number (KN) **(F)** for evaluation datasets.

**Figures 9A–C** shows the validation of the simulated KW, GY, and KN and their comparison against the measured values for the 81 wheat varieties in 2018–2019. The simulated and measured KW, GY, and KN values were highly consistent. We also validated the KW, GY, and KN for the 81 wheat varieties in 2019–2020 (**Figures 9D–F**). The fitted equations were y = 0.784x + 7.94, y = 0.985x - 375.08, and y = 0.369x + 13146.67, respectively. The RMSE were 2.602g TK^-1^, 689.772 kg ha^-1^, and 2,008.485 KN spike^-1^, respectively. The D values calculated from the measured and simulated values were 0.819, 0.802, and 0.613, respectively. The R^2^ were 0.5, 0.673, and 0.167 (P < 0.05), respectively.

### 3.5 Impact of wheat phenology, biomass, and KW under future climate change scenarios

**Figure 10:**
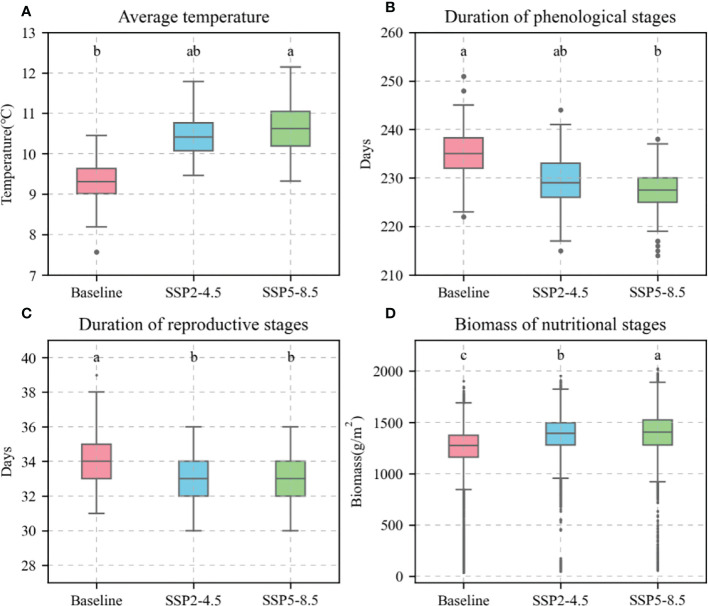
Average temperature, days, and biomass of 81 wheat varieties during phenological stages under different climate scenarios. **(A)**, average temperature; **(B)**, duration of phenological stages; **(C)**, duration of reproductive stages; **(D)**, biomass of nutritional stages. Red boxes represent baseline. Blue boxes represent SSP2-4.5. Green boxes represent SSP2-8.5. Different letters indicate significance at 0.05 level.

**Figure 11:**
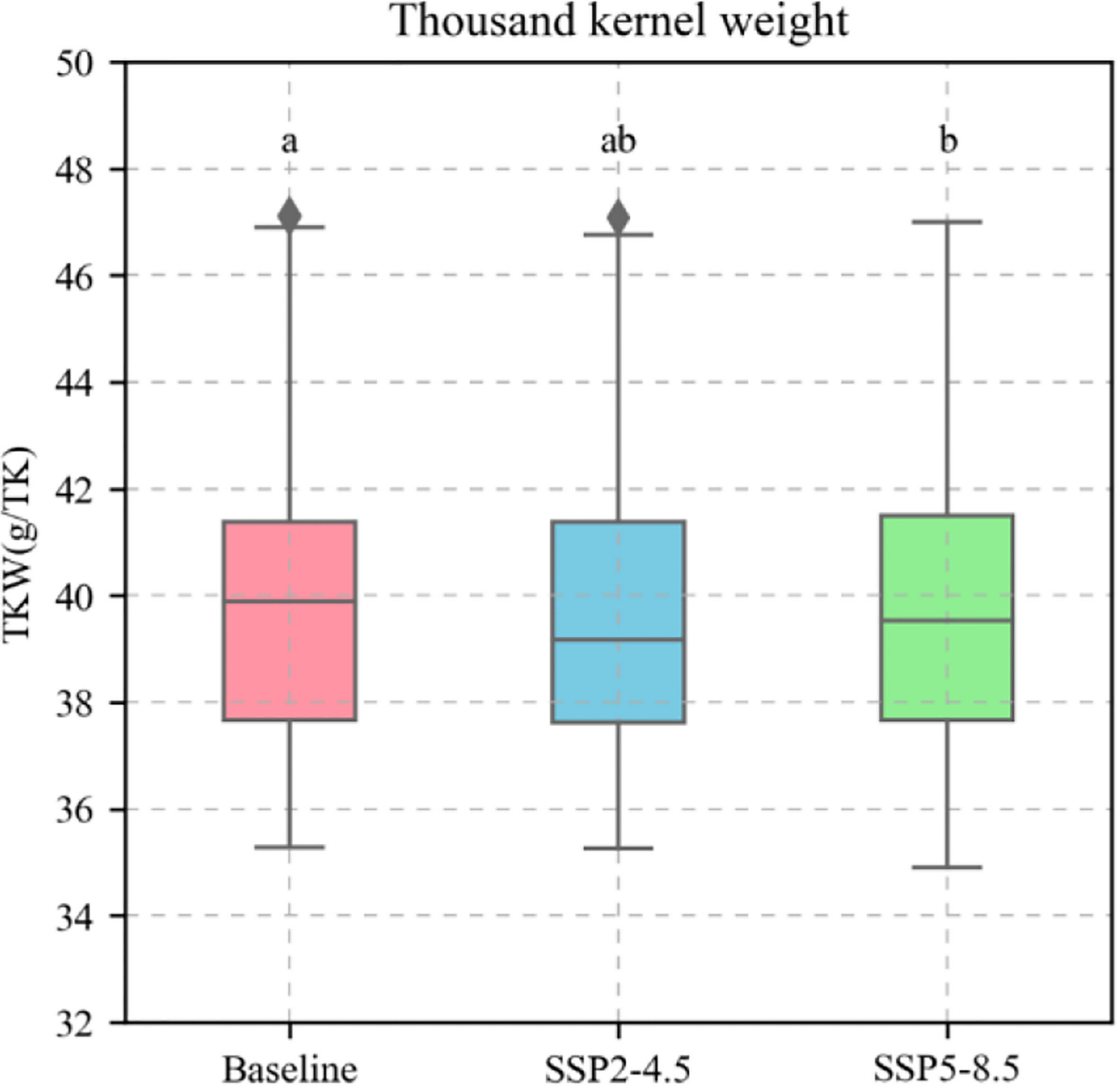
Thousand kernel weight (TKW) of 81 wheat varieties during phenological stages under different climate scenarios. The red boxes represent the Baseline; The blue boxes represent SSP2-4.5; The green boxes represent SSP2-8.5. The different letters indicate significance at the 0.05 level.

Based on the current sowing dates, varieties, and field managements at the Xinxiang station, the calibrated APSIM-Wheat model was used to simulate the phenological stages, biomass, and KW of winter wheat under the baseline (1991–2020), SPP2-4.5 (2031–2060), and SSP5-8.5 (2031–2060) climate scenarios. The simulation demonstrated a temporal increase in the average temperature during the winter wheat phenological stages, shortening of the latter, increasing biomass (reproductive stages) (**Figure 10A–D**), and decreasing KW (**Figure 11**). For the preceding climate scenarios, the average temperature for winter wheat were 9.3°C, 10.4°C, and 10.6°C, respectively; the mean phenological stages 235.21 d, 228.93 d, and 227.20 d, respectively; the mean reproductive stages were 33.83 d, 33.81 d, and 33.73 d, respectively; the mean biomass values for these reproductive stages were 1239.50 g m^−2^, 1374.99 g m^−2^, and 1386.21 g m^−2^, respectively; and the mean KW were 39.90 g TK-1, 39.58 g TK-1, and 39.65 g TK-1, respectively.

### 3.6 Responses of sowing date and allelic combinations to KW under future climate scenarios

**Figure 12:**
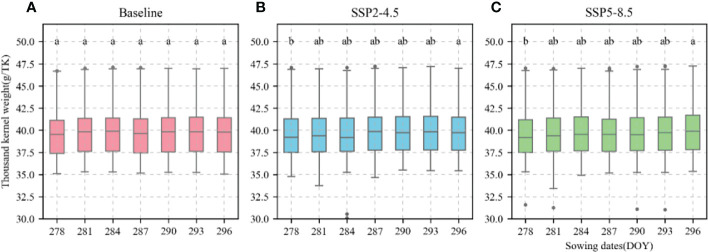
Thousand-kernel weight (TKW) of 81 wheat varieties at different sowing dates for different climate scenarios. **(A)**, Baseline; **(B)**, SSP2-4.5; **(C)**, SSP2-8.5. Current sowing date (DOY 284). Different letters indicate significance at 0.05 level.

**Figure 13:**
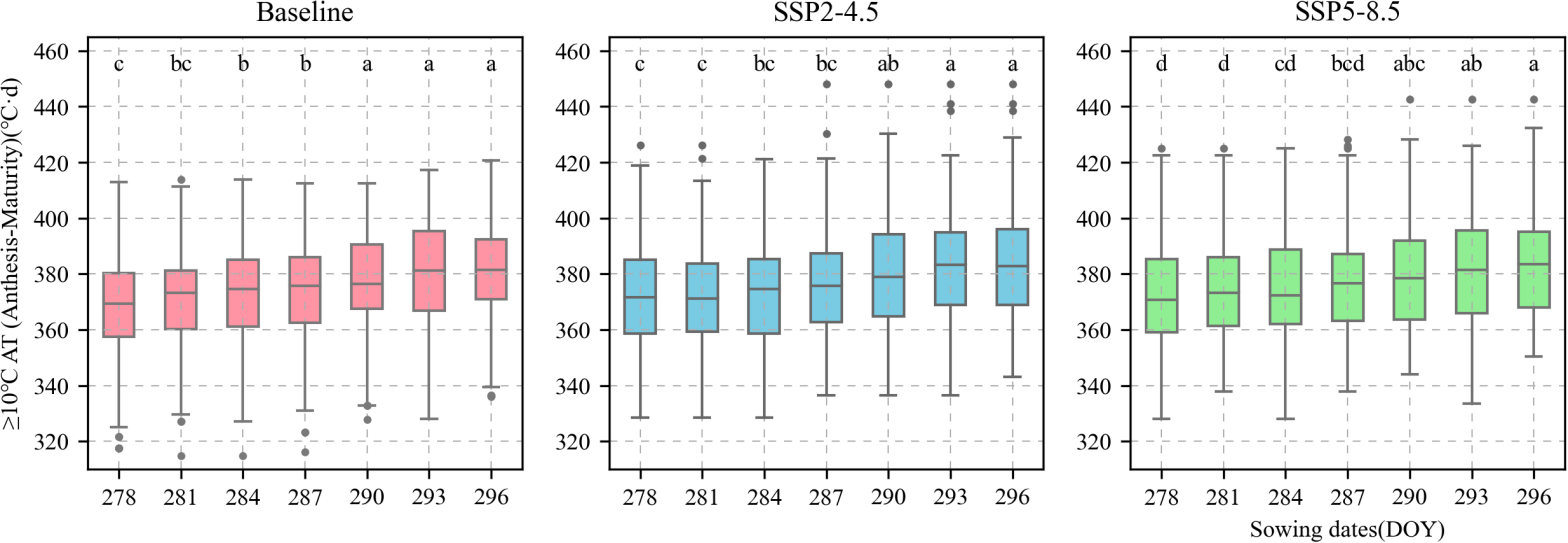
Accumulated temperature of ≥ 10°C from anthesis to maturity of 81 wheat varieties under different sowing dates. Current sowing date (DOY 284). Red boxes represent baseline. Blue boxes represent SSP2-4.5. Green boxes represent SSP2-8.5.

**Figure 14:**
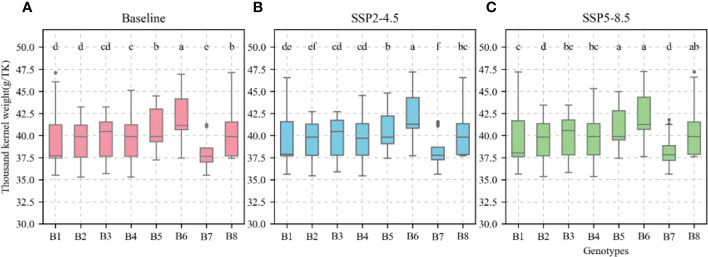
Thousand-kernel weight (TKW) of different allelic combination under different climatic scenarios at optimal sowing date. **(A)**, Baseline; **(B)**, SSP2-4.5; **(C)**, SSP2-8.5. Different letters indicate significance at 0.05 level.

The calibrated APSIM-Wheat model was used to simulate winter wheat KW under the aforementioned climate scenarios. ANOVA was then performed based on the simulation results. Allelic combination, climate scenario, and sowing date extremely significantly affected KW (P < 0.001) while the interaction between allelic combination and climate scenario significantly affected it (P < 0.05) (**Table 3**). **Figure 12** shows the non-significant effect of changing the sowing date on TKW and KW under SSP2-4.5 and SSP2-8.5 scenarios. The KW was 39.58 g TK^−1^ and 39.65 g TK^−1^ for the normal sowing period on October 11 (DOY 284), respectively. The average KW increased gradually from October 5 to October 23 (DOY 278–DOY 296; an average increase rate of 0.092 g TK^−1^ and 0.126 g TK^−1^, respectively). **Figure 13** shows that changing the sowing date for SSP2-4.5 and SSP2-8.5 scenarios had a significant effect on the accumulated temperature at the reproductive stages ≥10°C. The accumulated temperature at the reproductive stages (≥10°C) increased with delay in sowing, with an average increase rate of 1.57°C·d and 1.56°C·d, respectively. **Figure 14** shows the favorable allelic combination B6 *(TaCKX-D1b+ Hap-7A-1+ Hap-T+ Hap-6A-G+ Hap-6B-1+ H1g+ A1b)* with a KW of 40.13 g TK^−1^. For the SSP2-8.5 scenario, the favorable allelic combinations are B5, B6, and B8, with a KW of 40.53 g TK^-1^, 40.85 g TK^-1^, and 40.19 g TK^-1^, respectively, and the KW values of allelic combination B6 *(TaCKX-D1b+ Hap-7A-1+ Hap-T+ Hap-6A-G+ Hap-6B-1+ H1g+ A1b)*, from the first quartile (25%) to the third quartile (75%), from the box position, were higher than those of B5 and B8. Stable, favorable allelic combinations can mitigate the negative impact of climate change on wheat TKW.

**Table 3:** Variance analysis of effects of sowing date, genotype, and climate scenario and their interactions on thousand-kernel weight (TKW) in three growing seasons (2018–2020).

	df	Sum of Sq	Mean of Sq	F Value	Pr>F
Sowing dates	6	316	52.6	7.439***	<0.001
Genotype	7	9622	1374.5	194.289***	<0.001
Climate scenario	3	64	21.4	3.023*	0.02841
Sowing dates×Genotype	42	156	3.7	0.525	0.99516
Sowing dates×Climate scenario	18	131	7.3	1.026	0.42533
Genotype×Climate scenario	21	301	14.3	2.028**	0.00358
Sowing dates×Genotype×Climate scenario	126	187	1.5	0.21	1.00000

*** Indicates significance at the 0.001 level, ** Indicates significance at the 0.01 level, and * indicates significance at the 0.05 level

### 3.7 Values of the variety parameters for different allelic combinations

**Figure 15:**
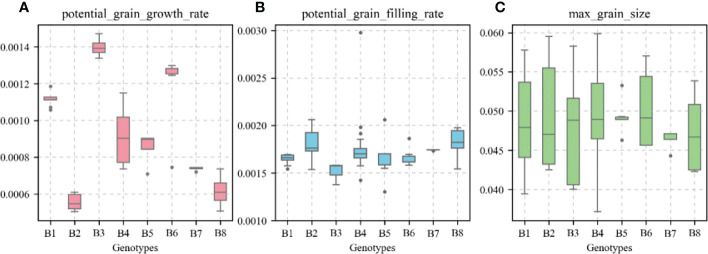
Values of variety parameters in the APSIM-Wheat model for different genotypes. **(A)**, potential grain growth rate; **(B)**, potential grain filling rate; **(C)**, max grain size.

**Figure 15** shows the values of the species-type parameters “potential_grain_growth_rate,” “potential_grain_filling_rate,” and “max_grain_size” for different allelic combinations in the APSIM-Wheat model. In the B6 high-KW allelic combination, “max_grain_size” had the highest value with a mean value of 0.0503; the “potential_grain_growth_rate” parameter had the highest value (top 25%) with a mean value of 0.00128; the “potential_grain_filling_rate” parameter had the median value (front 50%) with a mean value of 0.00167.

## 4 Discussion

### 4.1 Effects of the parameter sensitivity analysis on APSIM-Wheat

Parameter localization is a prerequisite for effective crop model use. Parameter sensitivity analysis facilitates targeted model parameter calibration (Kirui et al., 2022). Process-based physiological and ecological models simulate crop growth, development, and water and nutrient uptake. They can explore the dynamic relationships among atmosphere, crop, and soil and enable the prediction of crop responses to climate, genotype, farm management, and their interactions (Briak and Kebede, 2021). Prior studies focused on crop variety parameters. However, Zhao et al. (2014) reported that the APSIM-Wheat model includes other variables that may also affect the output. To minimize model uncertainty, researchers performed a sensitivity analysis of eight variety parameters and 33 ecotype parameters. Kernel weight (KW) was sensitive to the variety parameters “max_grain_size”, “potential_grain_growth_rate”, and “potential_grain_filling_rate”. This finding was consistent with the results of Zhao et al. (2014). Nevertheless, KW was also sensitive to the ecotype parameters “y_swdef_leaf”, “y_rue”, “eo_crop_factor_default”, “fr_lf_sen_rate”, and “node_sen_ rate”. Other studies reported contradictory results (He et al., 2015; Casadebaig et al., 2016). Broadening the parameter range reduces the uncertainty of the model and improves its performance at simulating winter wheat growth and development (Silva et al., 2021).

### 4.2 APSIM-Wheat parameter calibration and evaluation

Calibrating and evaluating APSIM-Wheat is the foundation for determining the optimal sowing time and allelic combination high KW in winter wheat under future climate scenarios (Ahmed et al., 2016). The present study validated the variety and ecotype parameters for KW and GY based on the validation of the model parameters phenological period, soil moisture content, nitrate nitrogen, and particle number. This analysis provides theoretical support for subsequent investigations of the source-sink relationships in wheat KW. **Figures 5**, **8** show that after calibration and evaluation, the APSIM-Wheat model simulated phenological period, soil moisture content, and nitrate nitrogen reasonably well. The R^2^ were 0.993, 0.124, and 0.286, respectively, and the RMSE were 7.588 d, 0.06 d, and 2.892 d, respectively. Hence, the APSIM-Wheat model lays a theoretical foundation for optimizing the parameters related to KW and GY. Substantial differences were observed in the sowing dates over 2018–2020, the weather conditions during the sowing periods, and the multi-year phenological data from the agrometeorological stations. Nonetheless, the calibrated and evaluated APSIM-Wheat model reflected the responses to different sowing dates. **Figure 9D** shows that the calibrated and evaluated APSIM-Wheat model accurately simulated KW (R^2^ = 0.575; RMSE = 3.076 g TK^-1^). The APSIM-Wheat model had an input of 209 varieties and accurately simulated the phenological period and the soil water-nitrogen balance. Thus, the calibrated and evaluated model reflected the responses to diverse allelic combinations and is an important tool for selecting optimal sowing dates and allelic combinations in future studies.

### 4.3 Effect of climate warming on wheat kernel weight

The APSIM-Wheat simulation-based study revealed that climate warming is an important cause of KW reduction in wheat. KW is influenced by biomass accumulation during vegetative growth in winter wheat. It is also constrained by grain size and filling rate (Almeida and Lidon, 2009; Liu et al., 2020). Juknys et al. (2017) reported that according to long-term phenological data, climate warming can shorten the nutritional growth stages of winter wheat and result in insufficient biomass accumulation during the pre-reproductive stages as well as inadequate KW during the post-reproductive stages. Jenner et al. (1993) demonstrated that elevated temperatures reduce KW by downregulating the enzymes biosynthesizing soluble starch. In this manner, high temperatures shorten grain filling. The APSIM-Wheat model simulation of the present study forecasted that the average temperature will increase and the phenological stages will decrease in future winter wheat growing seasons (**Figures 10A, B**). However, biomass will increase (**Figure 10D**). Hence, the KW source will increase and the sink (grain-filling period from flowering to maturity) influences the decrease in KW. Field and greenhouse experiments conducted by Xie et al. (2015) showed that faster and longer grain filling were associated with higher KW. In the present study, the APSIM-Wheat model parameters “max grain size”, “potential_grain_growth_rate”, and “potential_grain_filling_rate” reflected the post-flowering grain size and the grain filling and growth rates which determine KW. In APSIM-Wheat, variety parameters “max_grain_size”, “potential_grain_growth_rate”, and “potential_grain_filling_rate” reflected maximum grain size, potential growth rate, and filling rate under ideal climatic conditions, respectively, and are affected by climatic conditions (primarily temperature). In this study, the actual grain filling rate in APSIM-Wheat decelerated and the growth rate accelerated under the future climate scenario with an increase in the average daily temperature (**Figure 10A**). Lobell et al. (2012) reported that an increase in the average daily temperature increased the transpiration rate of the grains, leading to water loss and negatively affecting the grain filling rate. In addition, Barlow et al. (2015) reported that an increase in average daily temperature will increase the growth rate of the grain, leading to a reduction in the duration of grain filling. In this study, the reproductive growth phase of winter wheat was shortened under the future climate scenario (**Figure 10C**). Therefore, the deceleration of filling rate and shortening of filling duration caused by climate warming are responsible for the reduction in KW.

### 4.4 Effect of sowing date on wheat kernel weight

Slight changes in the sowing window have only a marginal impact on KW. Sowing date modification has the lowest cost of all farm management measures and is a key factor in KW. Sowing date is mainly under human control and its modulation may subject winter wheat to different weather factors such as temperature and accumulated temperature that affect KW. For example, Shah et al. (2020) stated that delaying the sowing date by four weeks lowered the accumulated temperature from a normal sowing time value of 514°C to a wintertime value of 226°C, negatively impacted early growth, and reduced KW. As the present study used a maize-wheat rotation system, the wheat had to be harvested in a timely manner to allow for maize sowing. Therefore, we used the APSIM-Wheat model to establish seven sowing windows that started on October 5 and were repeated every 3 d until October 23. A delayed sowing window slightly increased KW because the adjustments to the sowing window led to a slight increase in the accumulated temperature (**Figure 13**). For this reason, limited modifications to the sowing window resulted in slightly increased KW. Zheng et al. (2016) implemented the CSM-CERES-Wheat model in southern Khuzestan, Iran to adjust the sowing window to start on October 25 and repeat every 10 d until January 5. They found that fewer adjustments to the sowing window had a negligible impact on KW.

### 4.5 Relationship between variety parameter values and kernel weight alleleic combinations in the APSIM-Wheat model

The variety parameters and their relative importance in the APSIM-Wheat model are closely related to KW. In general, high KW is accompanied by the upregulation of functional alleles associated with favorable KW (Zhang et al., 2014; Chegdali et al., 2022; Tillett et al., 2022). The APSIM-Wheat model parameters “max_grain_size”, “potential_grain_ filling_rate”, and “potential_grain_growth_rate” had the strongest influences on wheat KW (Zhao et al., 2014). Our first-order and global parameter sensitivity analyses showed that “max_grain_size” had the greatest impact on wheat KW followed by “potential_grain_filling_rate” and “potential_grain_growth_rate”. Several studies demonstrated that “max_grain_size” adjusts the grain size, “potential_grain_filling_rate” adjusts the grain filling rate, and “potential_grain_growth_rate” adjusts the grain growth rate (Zhao et al., 2014; Xie et al., 2015; Kobata et al., 2018). The present study showed that the values for the parameters characteristic of winter wheat varieties with high KW were consistent with high KW allelic combinations. In the B6 high-KW allelic combination, “max_grain_size” had the highest value (**Figure 15**). This observation may be explained by upregulation of the advantageous A1b allele of the KW functional gene *TaGS5-A1* which promotes cell division and the formation of large kernels (Wang et al., 2016). In the B7 low-KW allelic combination, “max_grain_size” had one of the lowest values of all parameters (bottom 25%) (**Figure 15**). This finding may be explained by the upregulation of the advantageous A1a allele of the KW functional gene *TaGS5-A1*. An earlier study showed that the KW functional genes *TaSus1-7A* and *TaSus1-7B* encoding sucrose synthases have the advantageous alleles Hap-7A-1/2 and Hap-T that promote starch sediment biosynthesis and facilitate grain filling (Hou et al., 2014). In the B6 high-KW allelic combination, the “potential_grain_growth_rate” parameter had the highest value (top 25%), and the “potential_grain_filling_rate” parameter had the median value (front 50%) (**Figure 15**). This discovery may be explained by the upregulation of the advantageous allele Hap-6B-1 of the KW functional gene *TaGW2-6B*, which regulates the number of endosperm cells, promotes grain development, and is superior to the favorable allele “Hap-6B-2” (Qin et al., 2014).The foregoing reports revealed that the APSIM-Wheat model variety parameter values effectively captured KW expression in different allelic combinations.

### 4.6 Response of the kernel weight functional genes to climate warming

Determining the responses of KW functional genes to climate warming facilitates the identification of high-KW allelic combinations. Here, multivariate ANOVA revealed that allelic combinations and climate scenarios extremely significantly influenced TKW (P < 0.001) while their interaction significantly affected it (P < 0.05). Under different climate scenarios, the B6 allelic combination had higher KW and was stable. The B7 allelic combination had lower KW than the other allelic combinations. The preceding results suggest that the values of the variety parameter determining high KW were consistent with the high-KW allelic combinations. The variety parameter values were highest for the B6 allelic combination which included the KW functional genes *TaSus1-7A, TaSus1-7B, TaGW2-6B*, and *TaGS5-A1* and their favorable alleles Hap-7A-1, Hap-T, Hap-6B-1, and A1b, respectively. The B7 allelic combination had low variety parameter values but included the KW functional genes, *TaSus1-7A, TaSus1-7B, TaGW2-6A*, and *TaGW2-6B*, and their favorable alleles, Hap-7A-2, Hap-T, Hap-6A-A, and Hap-6B-2, respectively. A recent study revealed that in *TaSus1-7A*, Hap-7A-1 and Hap7A-2 had strong and weak effects on wheat KW, respectively (Sehgal et al., 2019). The favorable allele Hap-6B-1 of *TaGW2-6B* has a stronger impact on KW than the favorable allele Hap-6B-2. However, both had a greater effect on KW than *TaGW2-6A* (Hou et al., 2014). The favorable allele of TaSus1-7A, “Hap-1/2,” was measured in multiple environments with high TKW and stable expression of the trait (Qin et al., 2014).Therefore, the B6 allelic combination can achieve maximum KW. The present and preceding studies provide critical reference for breeding winter wheat and certain other cereal crops with high kernel weight under climate warning.

## 5 Conclusions

The present study demonstrated the responses of wheat KW to diverse allelic combinations under predicted climate warming and identified the genetic adaptations that will enable winter wheat to remain productive as atmospheric temperatures continue to increase. We conducted field trials and KASP genotyping on 209 wheat varieties in 2018–2020 and compiled a unique dataset comprising phenotype, genotype, climate, and soil data as well as on-farm management information. We assumed that wheat varieties with KN and flowering period NDVI in close proximity had other genetic factors near the KW-regulating gene. Hence, 81 wheat varieties with similar KN and flowering period NDVI were selected for calibration and evaluation by using the process-based APSIM-Wheat model. The model simulated TKW for eight allele combinations (81 wheat varieties), seven sowing dates, and two shared socioeconomic pathways (SSPs). It was determined that variety and ecotype parameters significantly influenced winter wheat KW, GY, and KN. Localized APSIM-Wheat parameters could accurately calibrate and evaluate these three yield metrics. The variety parameters and their relative importance in the APSIM-Wheat model were consistent with the expression of functional genes in allelic combinations. Therefore, the selected variety parameters revealed diverse allelic combinations affecting KW re-expression. The wheat phenological stages will be shortened and KW will be reduced (0.11-0.18 g TK^-1^) under future climate scenarios. Nevertheless, clarification of the responses of KW to diverse allelic combinations under projected climate warming scenarios may help ameliorate the negative impact of climate warming on TKW. Under SSP2-4.5, favorable allele combinations (TaCKX-D1b + Hap-7A-1 + Hap-T + Hap-6A-G + Hap-6B-1 + H1g + A1b) would increase KW by 0.34 g TK^-1^, Under SSP5-8.5, the favorable allele combinations (TaCKX-D1b + Hap-7A-1 + Hap-T + Hap-6A-G + Hap-6B-1 + H1g + A1b) would increase KW by 0.26 g TK^-1^. The results of the present study showed that favorable allelic combinations could help achieve high winter wheat TKW even under the projected climate warming scenarios. The present study provides valuable theoretical and practical reference for breeders in the development and selection of high-yield, thermotolerant wheat varieties with the aid of the APSIM-Wheat model. These novel wheat cultivars could improve wheat productivity, profitability, and water-use efficiency and help maintain food security. Future research should endeavor to incorporate other genetic loci contributing to TKW into the APSIM-Wheat model, enabling the construction of genetic based-model by strongly linking genetic information to crop models.

## Data availability statement

The original contributions presented in the study are included in the article/**Supplementary Material**. Further inquiries can be directed to the corresponding author.

## Author contributions

Conceptualization, KW, YH; formal analysis, KW, LS and YH; funding acquisition, YH; writing, KW, BZ and YH; writing-review and editing, YH. These authors contributed equally: KW and LS. All authors contributed to the article and approved the submitted version.
