# Supplementary material for: Responses of wheat kernel weight to diverse allelic combinations under projected climate change conditions
Source: Front Plant Sci. 2023 Mar 14;14:1138966. doi: 10.3389/fpls.2023.1138966 (PMC10043320; doi:10.3389/fpls.2023.1138966)
Supplement: Supplementary file 1 [file Table_1.docx]

Supplementary Material

Responses of wheat kernel weight to diverse allelic combinations under projected climate change conditions

**Keyi Wang^1^,** **Liping Shi^1#^, Bangyou Zheng^2^, Yong He^1*^**

^1^Institute of Environment and Sustainable Development in Agriculture, Chinese Academy of Agricultural Sciences, Beijing, 100081, PR China

^2^CSIRO Agriculture and Food, Queensland Biosciences Precinct, St Lucia, Queensland, 4067, Australia

***Correspondence:**

**Yong He**

**Email:** [**heyong01@caas.cn**](mailto:heyong01@caas.cn)

**TEL: +86-10-82109767**

# Supplementary Tables

**Supplementary Table 1.** The primer sequences and the amplification condition of each gene.

| Trait | Gene | FAM Primer (5′-3′) | HEX Primer (5′-3′) | Common Primer (5′-3′) | Amplification Conditions |
| --- | --- | --- | --- | --- | --- |
| kernel weight | TaCKX-D1 | GAAGGTGACCAAGTTCATGCTCGTCGATAGTCTCATGCATATGC | GAAGGTCGGAGTCAACGGATTATGCATGCATGCATGCGT | AACTTTTCACGGTGAACAG | Primer mixture included 46 μL ddH2O, 30 μL common primer (100 μM) and 12 μL of each tailed primer (100 μM). Assays were tested in 384-well format and set up as 5 μL reaction [2.2 μL DNA (10–20 ng/μL), 2.5 μL of 2XKASP master mixture and 0.056 μL primer mixture]. PCR cycling was performed using following protocol: hot start at 95 °C for 15 min, followed by ten touchdown cycles (95 °C for 20 s; touchdown 65 °C–1 °C per cycle 25 s) further followed by 30 cycles of amplification (95 °C for 10 s; 57 °C for 60 s). Extension step is unnecessary as amplicon is less than 120 bp. Plate was read in BioTek H1 system and data analysis was performed manually using Klustercaller software (version 2.22.0.5; LGC Hoddesdon, United Kingdom). |
|  | TaGASR7-A1 | GAAGGTGACCAAGTTCATGCTCACGGTAGAGGAGCCGGTTC | GAAGGTCGGAGTCAACGGAT-  TATAACTGCTCACCCCCCACC | ATATGTAGGGCAGGAAGGGC |  |
|  | TaSus1-7A | GAAGGTGACCAAGTTCATGCTGATTTGATCCATGCCCTCTC | GAAGGTCGGAGTCAACGGAT-  TGATTTGATCCATGCCCTCTT | CTGTCGTTCAACATCATTGTCTG |  |
|  | TaSus1-7B | GAAGGTGACCAAGTTCATGCTCAATTGCTTATGTTCTGTTGTATGG | GAAGGTCGGAGTCAACGGAT-  TCAATTGCTTATGTTCTGTTGTACAT | ATGGTTATGCTTGAATGGAAGAGC |  |
|  | TaGS5-A1 | GAAGGTGACCAAGTTCATGCTGTGCAATCTTGGACAAACATCAG | GAAGGTCGGAGTCAACGGAT-  TGTGCAATCTTGGACAAACATCAT | AGTGCTTTGTCAACAACAGATGC |  |
|  | TaGW2-6A | GAAGGTGACCAAGTTCATGCTTCCCGCTCCAGCTATCTGGTGAAC | GAAGGTCGGAGTCAACGGAT-  TTCCCGCTCCAGCTATCTGGTGAAA | TTCCCAGTCTTTGACATGTTCCGCC |  |
|  | TaGW2-6B | GAAGGTGACCAAGTTCATGCTTGAGATCCCGTGCAGTAGCTCG | GAAGGTCGGAGTCAACGGAT-  TTGAGATCCCGTGCAGTAGCTCA | TGGCGTGAGCTAGGGTTTGTTG |  |

**Supplementary Table 2.** Allelic combinations for kernel weight (A, 41 combinations)

| Haplotype | TaCKX-D1 | TaSus1-7A | TaSus1-7B | TaGW2-6A | TaGW2-6B | TaGASR7-A1 | TaGS5-A1 | Percent/% |
| --- | --- | --- | --- | --- | --- | --- | --- | --- |
| A1 | TaCKX-D1b | Hap-7A-1 | Hap-T | Hap-A | Hap-6B-1 | H1g | A1b | 26.09 |
| A2 | TaCKX-D1b | Hap-7A-1 | Hap-T | Hap-A | Hap-6B-1 | H1g | A1a | 10.63 |
| A3 | TaCKX-D1b | Hap-7A-1 | Hap-T | Hap-A | Hap-6B-1 | H1c | A1b | 10.14 |
| A4 | TaCKX-D1b | Hap-7A-2 | Hap-T | Hap-A | Hap-6B-1 | H1g | A1b | 6.76 |
| A5 | TaCKX-D1b | Hap-7A-1 | Hap-T | Hap-A | Hap-6B-2 | H1g | A1b | 5.80 |
| A6 | TaCKX-D1b | Hap-7A-1 | Hap-T | Hap-A | Hap-6B-2 | H1g | A1a | 5.31 |
| A7 | TaCKX-D1b | Hap-7A-1 | Hap-T | Hap-G | Hap-6B-2 | H1g | A1b | 4.35 |
| A8 | TaCKX-D1b | Hap-7A-1 | Hap-T | Hap-G | Hap-6B-1 | H1g | A1b | 3.86 |
| A9 | TaCKX-D1b | Hap-7A-2 | Hap-T | Hap-A | Hap-6B-1 | H1g | A1a | 2.90 |
| A10 | TaCKX-D1b | Hap-7A-2 | Hap-T | Hap-A | Hap-6B-2 | H1g | A1a | 2.42 |
| A11 | TaCKX-D1b | Hap-7A-1 | Hap-T | Hap-A | Hap-6B-2 | H1c | A1a | 2.42 |
| A12 | TaCKX-D1b | Hap-7A-1 | Hap-T | Hap-A | Hap-6B-2 | H1c | A1b | 1.93 |
| A13 | TaCKX-D1b | Hap-7A-1 | Hap-T | Hap-G | Hap-6B-1 | H1g | A1a | 1.45 |
| A14 | TaCKX-D1b | Hap-7A-2 | Hap-T | Hap-A | Hap-6B-1 | H1c | A1b | 0.97 |
| A15 | TaCKX-D1b | Hap-7A-2 | Hap-T | Hap-G | Hap-6B-1 | H1c | A1b | 0.97 |
| A16 | TaCKX-D1b | Hap-7A-2 | Hap-T | Hap-A | Hap-6B-2 | H1c | A1a | 0.97 |
| A17 | TaCKX-D1b | Hap-7A-1 | Hap-C | Hap-A | Hap-6B-2 | H1g | A1b | 0.97 |
| A18 | TaCKX-D1c | Hap-7A-1 | Hap-C | Hap-A | Hap-6B-2 | H1g | A1a | 0.97 |
| A19 | TaCKX-D1b | Hap-7A-1 | Hap-T | Hap-A | Hap-6B-4 | H1g | A1b | 0.48 |
| A20 | TaCKX-D1b | Hap-7A-1 | Hap-C | Hap-A | Hap-6B-1 | H1c | A1b | 0.48 |
| A21 | TaCKX-D1b | Hap-7A-1 | Hap-C | Hap-A | Hap-6B-1 | H1g/H1c | A1b | 0.48 |
| A22 | TaCKX-D1b | Hap-7A-2 | Hap-T | Hap-A | Hap-6B-1 | H1c | A1a | 0.48 |
| A23 | TaCKX-D1c | Hap-7A-2 | Hap-C | Hap-A | Hap-6B-2 | H1g | A1a | 0.48 |
| A24 | TaCKX-D1a | Hap-7A-3 | Hap-T | Hap-G | Hap-6B-2 | H1c | A1a | 0.48 |
| A25 | TaCKX-D1b | Hap-7A-2 | Hap-T | Hap-A | Hap-6B-2 | H1g | A1b | 0.48 |
| A26 | TaCKX-D1b | Hap-7A-3 | Hap-T | Hap-G | Hap-6B-2 | H1g | A1b | 0.48 |
| A27 | TaCKX-D1b | Hap-7A-1 | Hap-C | Hap-A | Hap-6B-1 | H1g | A1b | 0.48 |
| A28 | TaCKX-D1b | Hap-7A-3 | Hap-T | Hap-A | Hap-6B-1 | H1g | A1b | 0.48 |
| A29 | TaCKX-D1a | Hap-7A-2 | Hap-T | Hap-A | Hap-6B-2 | H1g | A1a | 0.48 |
| A30 | TaCKX-D1b | Hap-7A-1 | Hap-T/Hap-C | Hap-A | Hap-6B-2 | H1g | A1a | 0.48 |
| A31 | TaCKX-D1b | Hap-7A-2 | Hap-T | Hap-G | Hap-6B-1 | H1g | A1a | 0.48 |
| A32 | TaCKX-D1b | Hap-7A-1 | Hap-T | Hap-G | Hap-6B-1 | H1c | A1b | 0.48 |
| A33 | TaCKX-D1b | Hap-7A-2 | Hap-T | Hap-G | Hap-6B-2 | H1c | A1b | 0.48 |
| A34 | TaCKX-D1b | Hap-7A-1 | Hap-T | Hap-G | Hap-6B-2 | H1g | A1a | 0.48 |
| A35 | TaCKX-D1b | Hap-7A-1 | Hap-T | Hap-A | Hap-6B-1 | H1g | A1a/A1b | 0.48 |
| A36 | TaCKX-D1b | Hap-7A-2 | Hap-T | Hap-A | Hap-6B-2 | H1c | A1b | 0.48 |
| A37 | TaCKX-D1b | Hap-7A-1 | Hap-T | Hap-G | Hap-6B-1 | H1c | A1a | 0.48 |
| A38 | TaCKX-D1b | Hap-7A-4 | Hap-T | Hap-A | Hap-6B-2 | H1c | A1a | 0.48 |
| A39 | TaCKX-D1b | Hap-7A-1 | Hap-T/Hap-C | Hap-G | Hap-6B-2 | H1g | A1b | 0.48 |
| A40 | TaCKX-D1b | Hap-7A-1 | Hap-T | Hap-G/Hap-A | Hap-6B-1 | H1g | A1b | 0.48 |
| A41 | TaCKX-D1b | Hap-7A-1 | Hap-C | Hap-G | Hap-6B-1 | H1g | A1b | 0.48 |

**Supplementary Table 3.** On-farm management of winter wheat during the 2017-2018 and 2018-2019 growing seasons

| Field on-farm management | Growing seasons | |
| --- | --- | --- |
|  | 2017-2018 | 2018-2019 |
| Sowing date | 2017-10-15 | 2018-10-15 |
| Sowing density (million plants /ha) | 150 | 150 |
| Row space (cm) | 20 | 20 |
| Date of irrigation / Amount of irrigation (mm) | 2018-03-12 / 50 | 2019-03-12 / 50 |
|  | 2018-04-08 / 10 | 2019-04-13 / 50 |
|  | 2018-05-06 / 40 | 2019-05-03 / 50 |
|  | 2018-05-13 / 10 | 2019-05-16 / 50 |
| Date of fertilizer / Amount of fertilizer  (kg/ha) | 2017-10-14 / N 67.5 + P 105 + K 120 | 2018-10-14 / N 67.5 + P 105 + K 120 |
|  | 2018-03-12 / N 67.5 | 2019-03-12 / N 67.5 |
|  | 2018-04-08 / N 67.5 | 2019-04-03 / N 67.5 |
|  | 2018-05-13 / N 67.5 | 2019-05-16 / N 67.5 |

Notes: N, nitrogen fertilizer; P, phosphate fertilizer; K, potash fertilizer

**Supplementary Table 4.**  Characterization of physicochemical soil properties at different soil layers.

| Sowing depth (cm) | 0-20 | 20-40 | 40-60 | 60-80 | 80-100 |
| --- | --- | --- | --- | --- | --- |
| Clay (%) | 6.75 | 6.41 | 10.19 | 10.16 | 8.22 |
| Silt (%) | 69.22 | 66.91 | 69.96 | 73.44 | 75.74 |
| Sand (%) | 23.53 | 26.69 | 19.85 | 16.41 | 16.05 |
| Wilting point (cm^3^/cm^3^) | 0.16 | 0.16 | 0.18 | 0.18 | 0.17 |
| Field capacity (cm^3^/cm^3^) | 0.34 | 0.29 | 0.32 | 0.3 | 0.31 |
| Saturated water capacity (cm^3^/cm^3^) | 0.45 | 0.4 | 0.42 | 0.36 | 0.38 |
| Bulk density (g/cm^3^) | 1.58 | 1.6 | 1.55 | 1.42 | 1.45 |

**Supplementary Table 5.** Variety and ecotype parameters used in parameter sensitivity analysis via the Agricultural Production Systems Simulator (APSIM-Wheat) model.

| **Type** | **Abbreviation** | **Definition** | **Unit** | **Lower limit** | **Upper limit** |
| --- | --- | --- | --- | --- | --- |
| ecotype | y_swdef_leaf | Leaf water stress factor | ---- | 0.5 | 1 |
|  | y_swdef_fix | Fixation water stress factor | ---- | 0.5 | 1 |
|  | y_swdef_pheno_flowering | Flowering water stress factor | ---- | 0.7 | 1 |
|  | sfac_slope | Soil water factor slope | ---- | -0.1375 | -0.1125 |
|  | sw_fac_max | Maximum soil water factor | ---- | 1.0125 | 1.2375 |
|  | y_rue | Photosynthetic rate of use efficiency | g CO_2_/m^2^/d | 1.054 | 1.426 |
|  | y_stress_photo | Photosynthetic stress factor | ---- | 0.7 | 1 |
|  | oxdef_photo | Photosynthetic oxygen deficit factor | ---- | 0.8 | 1 |
| variety | minimum_grain_n_filling_rate | Minimum grain nitrogen filling rate | g N/day | 0.0000135 | 0.0000165 |
|  | sla_min | Minimum specific leaf area | m²/kg | 16200 | 19800 |
|  | specific_root_length | Specific root length | cm/kg | 94500 | 115500 |
|  | min_tpla | Minimum temperature for photosynthesis | ℃ | 4.5 | 5.5 |
|  | lai_sen_light | Leaf area index sensitivity to light | ---- | 6.3 | 7.7 |
|  | node_sen_rate | Node senescence rate | ---- | 40 | 90 |
|  | n_conc_crit_root | Critical nitrogen concentration in roots | g/kg | 0.01 | 0.06 |
|  | n_conc_max_root | Maximum nitrogen concentration in roots | g/kg | 0.02 | 0.06 |
|  | sen_light_slope | Light slope for senescence | ---- | 0.09 | 0.11 |
|  | meal_n_sen_conc | Meal nitrogen concentration for senescence | g/kg | 0.0126 | 0.0154 |
|  | initial_tpla | Initial temperature for photosynthesis | ℃ | 150 | 500 |
|  | initial_root_depth | Initial root depth | m | 50 | 300 |
|  | n_conc_min_grain | Minimum nitrogen concentration in grain | g/kg | 0.005 | 0.03 |
|  | fr_lf_sen_rate | Senescence rate for leaf fractions | ---- | 0.005 | 0.05 |
|  | y_node_app_rate | Apparent node number rate | ---- | 66.5 | 123.5 |
|  | y_leaves_per_node | Number of leaves per node | ---- | 1 | 10 |
|  | n_conc_min_root | Minimum nitrogen concentration in roots | g/kg | 0.009 | 0.011 |
|  | n_conc_min_grain | Minimum nitrogen concentration in grain | g/kg | 0.005 | 0.03 |
|  | n_conc_crit_grain | Critical nitrogen concentration in grain | g/kg | 0.027 | 0.033 |
|  | n_conc_max_grain | Maximum nitrogen concentration in grain | g/kg | 0.027 | 0.033 |
|  | n_sen_conc | Nitrogen sensitivity concentration | g/kg | 0.045 | 0.055 |
|  | root_n_sen_conc | Root nitrogen sensitivity concentration | g/kg | 0.045 | 0.055 |
|  | leaf_n_sen_conc | Leaf nitrogen sensitivity concentration | g/kg | 0.045 | 0.055 |
|  | stem_n_sen_conc | Stem nitrogen sensitivity concentration | g/kg | 0.00225 | 0.00275 |
|  | pod_n_sen_conc | Pod nitrogen sensitivity concentration | g/kg | 0.0063 | 0.0077 |
|  | eo_crop_factor_default | Evaporation crop factor default | ---- | 1 | 2 |
|  | N_fact_photo | Nitrogen photo-synthetic factor | ---- | 1 | 20 |
|  | N_fact_expansion | Nitrogen expansion factor | ---- | 0 | 5 |
|  | N_fact_grain | Nitrogen grain factor | ---- | 0 | 5 |
|  | grains_per_gram_stem | Grains per gram stem | ---- | 0 | 40 |
|  | potential_grain_growth_rate | Potential grain growth rate | ---- | 0.0005 | 0.0015 |
|  | max_grain_size | Maximum grain size | ---- | 0 | 0.055 |
|  | potential_grain_filling_rate | Potential grain filling rate | ---- | 0.00225 | 0.00375 |

**Supplementary Table 6.** Calibrated phenological parameters of APSIM-Wheat model

| Parameter | Definition | Initial value | Calibrated value |
| --- | --- | --- | --- |
| photop_sens | photoperiod sensitivity | 1.5-4.5 | 2.75 |
| vern_sens | vernalization sensitivity | 1.2-3.6 | 3.53 |
| tt_floral_initiation | thermal time for floral initiation | 349-610 | 591 |
| tt_start_grain_fill | thermal time for start of grain filling | 322-577 | 532 |

**Supplementary Table 7.** Calibrated soil parameters of APSIM-Wheat model

| Sowing depth (cm) | 0-10 | 10-20 | 20-30 | 30-40 | 40-60 | 60-80 |
| --- | --- | --- | --- | --- | --- | --- |
| Wilting point (cm^3^/cm^3^) | 0.126 | 0.116 | 0.127 | 0.122 | 0.137 | 0.127 |
| Field capacity (cm^3^/cm^3^) | 0.285 | 0.215 | 0.203 | 0.185 | 0.185 | 0.265 |
| Saturated water capacity (cm^3^/cm^3^) | 0.312 | 0.31 | 0.303 | 0.304 | 0.315 | 0.385 |
| Bulk density (g/cm^3^) | 1.475 | 1.319 | 1.253 | 1.22 | 1.301 | 1.42 |

**Supplementary Table 8.** Calibrated parameters linked to grain yield (GY), thousand grain weight (TGW) and kernel number per spike (KNS) of APSIM-Wheat model

| Type | Abbreviation | Definition | Range |
| --- | --- | --- | --- |
| ecotype | eo_crop_factor_default | Evaporation crop factor default | 0.4-1.5 |
| variety | node_sen_rate | Node senescence rate | 40-90 |
|  | fr_lf_sen_rate | Senescence rate for leaf fractions | 0.02-0.05 |
|  | y_rue | Photosynthetic rate of use efficiency, g CO_2_/m^2^/day | 1.24-1.43 |
|  | y_node_app_rate | Apparent node number rate | 66-124 |
|  | grains_per_gram_stem | Grains per gram stem | 10-40 |
|  | potential_grain_growth_rate | Potential grain growth rate | 0.0005-0.0015 |
|  | potential_grain_filling_rate | Potential grain filling rate | 0.001-0.005 |
|  | max_grain_size | Maximum grain size | 0.02-0.06 |
